# The expression and methylation of *PITX* genes is associated with the prognosis of head and neck squamous cell carcinoma

**DOI:** 10.3389/fgene.2022.982241

**Published:** 2022-09-20

**Authors:** Yaqiong Zhao, Jie Zhao, Mengmei Zhong, Qian Zhang, Fei Yan, Yunzhi Feng, Yue Guo

**Affiliations:** ^1^ Department of Stomatology, The Second Xiangya Hospital, Central South University, Changsha, China; ^2^ Hunan Key Laboratory of Oral Health Research and Hunan 3D Printing Engineering Research Center of Oral Care and Hunan Clinical Research Center of Oral Major Diseases and Oral Health and Xiangya Stomatological Hospital and Xiangya School of Stomatology, Central South University, Changsha, China

**Keywords:** PITX gene family, HNSC, DNA Methylation, prognosis, Bioinformatic analysis

## Abstract

**Background:** The *PITX* gene family, comprising *PITX1*, *PITX2*, and *PITX3*, is critical in organogenesis and has been evolutionary conserved in animals. *PITX* genes are associated with the advanced progression and poor prognosis of multiple cancers. However, the relationship between the *PITX* genes and head and neck squamous cell carcinoma (HNSC) has not been reported.

**Methods:** We used data from The Cancer Genome Atlas (TCGA) to analyze the association between *PITX* mRNA expression and clinicopathological parameters of patients with HNSC. The prognostic value of *PITX* genes was evaluated using the Kaplan-Meier plotter. Multivariate Cox analysis was used to screen out prognosis-associated genes to identify better prognostic indicators. The potential roles of *PITX1* and *PITX2* in HNSC prognosis were investigated using the protein-protein interaction (PPI) network, Gene Ontology (GO) analysis, and the Kyoto Encyclopedia of Genes and Genomes (KEGG) analysis. The correlation between *PITX1* and *PITX2* expression or methylation and immune cell infiltration was evaluated using the tumor-immune system interaction database (TISIDB). MethSurv was used to identify DNA methylation and its effect on HNSC prognosis.

**Results:** *PITX* genes expression was correlated with different cancers. *PITX1* and *PITX2* expression was lower in the patients with HNSC. In HNSC, *PITX1* expression was significantly related to the clinical stage, histologic grade, and N stage, while *PITX2* expression was only significantly related to the histologic grade. The high expression of *PITX3* was significantly related to the histologic grade, T stage, and N stage. Survival analysis revealed that *PITX* genes had prognostic value in HNSC, which was supported by multivariate Cox analysis. PPI network and enrichment analysis showed that the genes interacting with *PITX1* and *PITX2* belonged predominantly to signaling pathways associated with DNA binding and transcription. Of the CpG DNA methylation sites in *PITX1* and *PITX2*, 28 and 22 were related to the prognosis of HNSC, respectively. Additionally, *PITX1* and *PITX2* expression and methylation was associated with tumor-infiltrating lymphocytes (TILs).

**Conclusion:** The *PITX* genes were differentially expressed in patients with HNSC, highlighting their essential role in DNA methylation and tumor-infiltrating immune cell regulation, as well as overall prognostic value in HNSC.

## Introduction

Head and neck squamous cell carcinoma (HNSC) is the most common cancer of the head and neck. It originates from the squamous epithelium of the oral cavity, oropharynx, larynx, and hypopharynx (Solomon et al., 2018). It is the sixth most common cancer worldwide, with an incidence of ∼600,000 new cases each year, which is predicted to rise to 1.08 million new cases per year by 2030 (an increase of 30%) (Bray et al., 2018; Ferlay et al., 2019; Jiang et al., 2022). The early symptoms of HNSC are not obvious, meaning that the majority of patients are diagnosed at later stages of the disease, which is a serious threat to human health. At present, HNSC is primarily treated using resection, radiation, and systemic therapy; however, these strategies may lead to complications and poor long-term outcomes (Solomon et al., 2018). Accordingly, the 5-years survival rate for patients with HNSC remains less than 50%. Abnormal gene expression may be involved in tumorigenesis and is associated with the prognosis of HNSC patients (Zhang and Gao, 2021). In the past decade, elucidation of the molecular genetic landscape of HNSC has revealed new opportunities for therapeutic intervention, with a particular focus on immunotherapy. For instance, the immune checkpoint inhibitor pembrolizumab has been trialed as a first-line systemic treatment of HNSC (Saâda-Bouzid et al., 2017). Besides, detailed evaluation of the molecular characteristics of HNSC and immune profiling suggest that the inclusion of prognostic and predictive biomarkers in clinical studies may overcome the obstacles of targeted therapy and prolong the survival time of patients with HNSC (Oliva et al., 2019; Johnson et al., 2020). Hence, effective prognostic indicators for HNSC are urgently needed.

The *PITX* gene family belongs to the group of homeobox genes, which are highly conserved in all animals through evolution. To date, three *PITX* paralogs have been identified in mammalian cells: *PITX1*, *PITX2*, and *PITX3* (Tran and Kioussi, 2021). Due to the early expression of *PITX* genes during embryonic development, their role has been mainly studied in the context of tissue and organ formation (Angotzi et al., 2008). *PITX1* plays a role in pituitary development and hindlimb tissue configuration (Lanctôt et al., 1999). Meanwhile, *PITX2* regulates the development of the pituitary, the face, teeth, skeletal muscle, the heart, and the intestine (Shih et al., 2007). Finally, *PITX3* monitors dopaminergic neuronal function in the substantia nigra and during lens development. Recently, the *PITX* genes were shown to be involved in tumorigenesis. The expression of *PITX* activators is deregulated in some human malignancies, including hepatocellular carcinoma (Tai et al., 2016), lung cancer (Chen et al., 2007), prostate cancer (Poos et al., 2022), and cutaneous malignant melanoma (Osaki et al., 2013). *PITX1* has been reported as a candidate tumor suppressor gene and a possible biomarker for predicting the chemical sensitivity of HNSC in humans (Takenobu et al., 2016). A recent study of *PITX2* and an adjacent long non-coding RNA (lncRNA) methylation site, showed that hypermethylation was associated with improved survival in an HNSC cohort (Sailer et al., 2016). Moreover, *PITX3* DNA methylation has proved to be an independent prognostic biomarker for overall survival (OS) in patients with HNSC and could potentially assist with risk-group assignment and subsequent treatment stratification (Sailer et al., 2017b). However, the role of *PITX* gene expression and methylation in the infiltration of immune cells into the tumor and the prognosis of HNSC has not been described.

Here, we used a bioinformatics approach to elucidate the relationship between *PITX* genes and the oncologic characteristics of patients with HNSC as well as assess their ability to predict the prognosis of HNSC. We used The Cancer Genome Atlas (TCGA) to analyze the expression of different *PITX* transcription factors in patients with HNSC, in order to determine their expression pattern, potential mechanism of action, DNA methylation status, and their relationship with immune infiltration and the prognosis of HNSC.

## Materials and methods

### Raw data acquisition and processing

TCGA has profiled and analyzed a large collection of clinical and molecular data from over 10,000 tumor patients across 33 different tumor types (Weinstein et al., 2013; Colaprico et al., 2016). Transcriptome RNA sequencing (RNA-seq) data were extracted from TCGA (https://portal.gdc.cancer.gov/) for 33 cancers, which included: adrenocortical carcinoma (ACC); bladder urothelial carcinoma (BLCA); breast invasive carcinoma (BRCA); cervical squamous cell carcinoma and endocervical adenocarcinoma (CESC); cholangiocarcinoma (CHOL); colon adenocarcinoma (COAD); lymphoid neoplasm diffuse large B-cell lymphoma (DLBC); esophageal carcinoma (ESCA); glioblastoma multiforme (GBM); head and neck squamous cell carcinoma (HNSC); kidney chromophobe (KICH); kidney renal clear cell carcinoma (KIRC); kidney renal papillary cell carcinoma (KIRP); acute myeloid leukemia (LAML); brain lower grade glioma (LGG); liver hepatocellular carcinoma (LIHC); lung adenocarcinoma (LUAD); lung squamous cell carcinoma (LUSC); mesothelioma (MESO); ovarian serous cystadenocarcinoma (OV); pancreatic adenocarcinoma (PAAD); pheochromocytoma and paraganglioma (PCPG); prostate adenocarcinoma (PRAD); rectum adenocarcinoma (READ); sarcoma (SARC); skin cutaneous melanoma (SKCM); stomach adenocarcinoma (STAD); testicular germ cell tumors (TGCT); thyroid carcinoma (THCA); thymoma (THYM); uterine corpus endometrial carcinoma (UCEC); uterine carcinosarcoma (UCS) and uveal melanoma (UVM). RNA-seq data from 502 HNSC tissues and 44 normal tissues were downloaded from TCGA. The RNA-seq data and the corresponding patient clinical information (Workflow Type: HTSeq-FPKM) were acquired using the UCSC Xena tool (https://xenabrowser.net/datapages/). The level 3 HTSeq-FPKM data were transformed to log2 for the following analysis.

### Survival analysis and *PITX* genes expression

The Kaplan-Meier plotter (http://kmplot.com/analysis/index.php?p=service) is an online drawing tool used to evaluate the influence of 54,685 genes on survival by mining information from 10,471 samples. In this study, we used the Kaplan-Meier plotter to determine the prognostic value of *PITX* gene expression levels in predicting OS and relapse free survival (RFS) of patients with HNSC. A difference was considered statistically significant when the *p*-value was < 0.05. Univariate Cox proportional hazard regression analysis was conducted to screen the differentially expressed *PITX* genes significantly associated with OS of HNSC. Then, the differentially expressed *PITX* genes with a *p*-value of less than 0.1 were further identified by multivariate Cox proportional hazard regression. Genes with a *p*-value of less than 0.05 (according to the multivariate Cox proportional hazard regression analysis) were identified as prognosis-related genes.

### Analysis of the biological function of *PITX* genes

GeneMANIA is a flexible web interface used to construct protein-protein interaction (PPI) networks, generate hypotheses on gene function, and explore gene lists, while prioritizing genes (Warde-Farley et al., 2010). In this study, we used GeneMANIA to visualize the gene network and predict the functions of the co-expressed PITX1 and PITX2 genes. Gene Ontology (GO) enrichment and Kyoto Encyclopedia of Genes and Genomes (KEGG) pathway analysis of co-expressed genes were performed using the “ClusterProfiler” package (V 3.14.3) and visualized using the “ggplot2” package (V 3.3.3) (Xu et al., 2012).

### DNA methylation of prognosis-associated *PITX1* and *PITX2* genes

The MethSurv database (https://biit.cs.ut.ee/methsurv/) was used to analyze the DNA methylation sites of *PITX1* and *PITX2* in TCGA. Moreover, the prognostic value of *PITX1* and *PITX2* CpG methylation sites was evaluated using OS as the survival outcome measure.

### Tumor-immune estimation resource (TIMER) database analysis

TIMER 2.0 (http://timer.comp-genomics.org/) is a web server used for the analysis of infiltrating immune cells and their clinical impact (Li et al., 2020). In this study, the “gene” module was used to evaluate the relationship between *PITX* gene expression and immune cell infiltration. We chose *PITX1* or *PITX2* as input, while B cells, CD8^+^ T cells, CD4^+^ T cells, neutrophils, macrophages, and dendritic cells (DCs), were selected as the immune cell types (Li et al., 2016).

### Tumor-immune system interaction database (TISIDB) analysis

TISIDB (http://cis.Hku.hk/TISIDB/) is an online web integrated repository portal for tumor-immune system interactions (Ru et al., 2019). In this study, we used the TISIDB to determine the relationships between the abundance of tumor-infiltrating lymphocytes (TILs) and the expression or methylation of *PITX1* and *PITX2* across human cancers. Based on the gene expression profile, the relative abundance of TILs was inferred using gene set variation analysis. The correlations between the expression and methylation of *PITX1* and *PITX2* genes and TILs were measured using Spearman’s test.

### Statistical analysis

All statistical analysis were performed using R software (V 4.2.0) (https://www.r-project.org/) and the R package ggplot2 was used to visualize gene expression differences. The Wilcoxon rank-sum test was used to determine the differences between tumor tissues and adjacent normal tissues. The associations between *PITX* gene expression and clinicopathological parameters were measured using the Student’s t-test.

## Results

### The expression of *PITX* gene family members in different cancers

**FIGURE 1:**
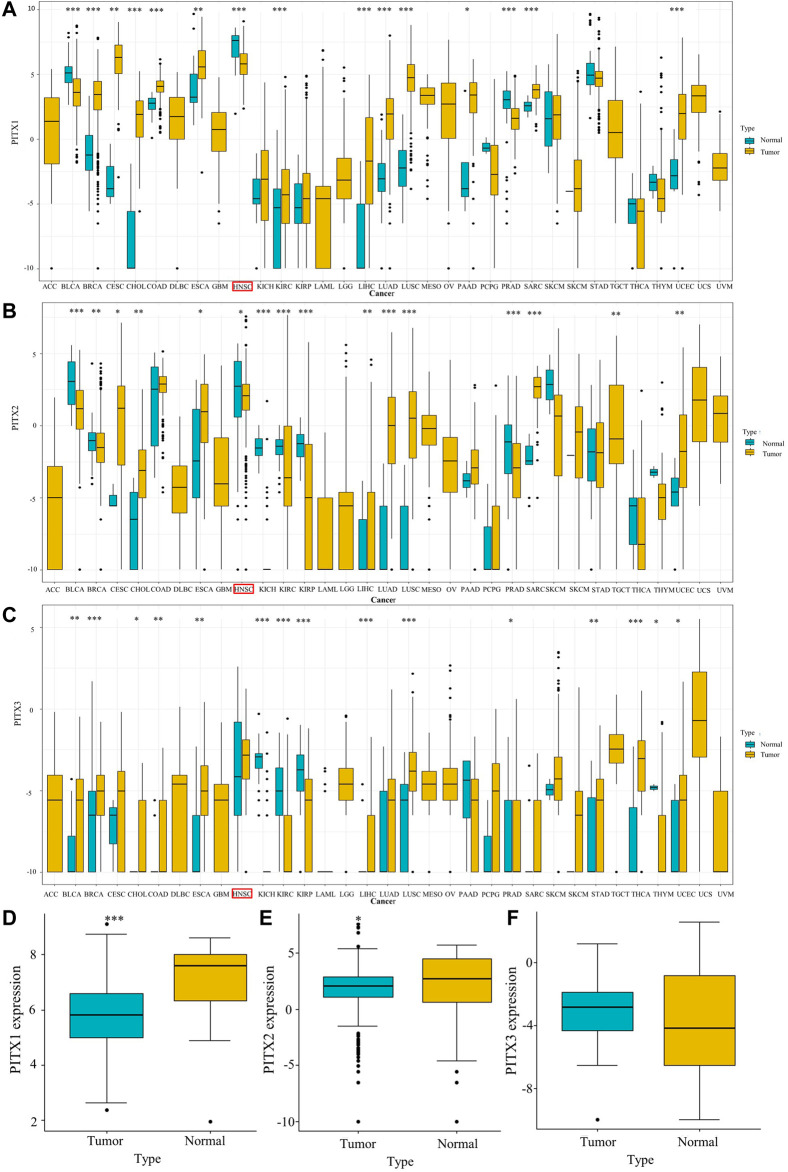
Expression of PITX genes in HNSC and normal tissue. **(A)** *PITX1*, **(B)** *PITX2*, and **(C)** *PITX3* expression levels in different tumor types from TCGA. **(D)** *PITX1*, **(E)** *PITX2*, and **(F)** *PITX3* expression difference in unpaired HNSC samples. *p* ≥ 0.05; *, *p* < 0.05; **, *p* < 0.01; ***, *p* < 0.001. ACC, adrenocortical carcinoma; BLCA, bladder urothelial carcinoma; BRCA, breast invasive carcinoma; CESC, cervical squamous cell carcinoma and endocervical adenocarcinoma; CHOL, cholangiocarcinoma; COAD, colon adenocarcinoma; DLBC, lymphoid neoplasm diffuse large B-cell lymphoma; ESCA, esophageal carcinoma; GBM, glioblastoma multiforme; HNSC, head and neck squamous cell carcinoma; KICH, kidney chromophobe; KIRC, kidney renal clear cell carcinoma; KIRP, kidney renal papillary cell carcinoma; LAML, acute myeloid leukemia; LGG, brain lower grade glioma; LIHC, liver hepatocellular carcinoma; LUAD, lung adenocarcinoma; LUSC, lung squamous cell carcinoma; MESO, mesothelioma; OV, ovarian serous cystadenocarcinoma; PAAD, pancreatic adenocarcinoma; PCPG, pheochromocytoma and paraganglioma; PRAD, prostate adenocarcinoma; READ, rectum adenocarcinoma; SARC, sarcoma; SKCM, skin cutaneous melanoma; STAD, stomach adenocarcinoma; TGCT, testicular germ cell tumors; THCA, thyroid carcinoma; THYM, thymoma; UCEC, uterine corpus endometrial carcinoma; UCS, uterine carcinosarcoma; UVM, uveal melanoma.

To understand whether the expression of *PITX* genes correlates with cancer, we evaluated *PITX1*, *PITX2*, and *PITX3* mRNA expression in different human tumors (33 cancer types) and adjacent normal tissues using TCGA data. The results showed that *PITX1* mRNA expression was associated with BLCA, BRCA, CESC, CHOL, COAD, ESCA, HNSC, KIRC, LIHC, LUAD, LUSC, PAAD, PRAD, READ, and UCEC (Figure 1A). The expression of *PITX2* was linked to BLCA, BRCA, CESC, CHOL, ESCA, HNSC, KICH, KIRC, KIRP, LIHC, LUAD, LUSC, PRAD, READ, THCA, and UCEC (Figure 1B). The expression of *PITX3* was related to BLCA, BRCA, CHOL, COAD, ESCA, KICH, KIRC, KIRP, LIHC, LUSC, PRAD, STAD, THCA, THYM, and UCEC (Figure 1C). Low expression of *PITX1* (*p* < 0.001, Figure 1D) and *PITX2* (*p* < 0.05, Figure 1E) was observed in HNSC tumor tissues compared with normal tissues in unpaired specimens. Meanwhile, the expression of *PITX3* was higher in HNSC than in normal samples, but this difference was not statistically significant (*p* > 0.05, Figure 1F).

### Expression of *PITX* genes and clinicopathological characteristics of HNSC patients

**FIGURE 2:**
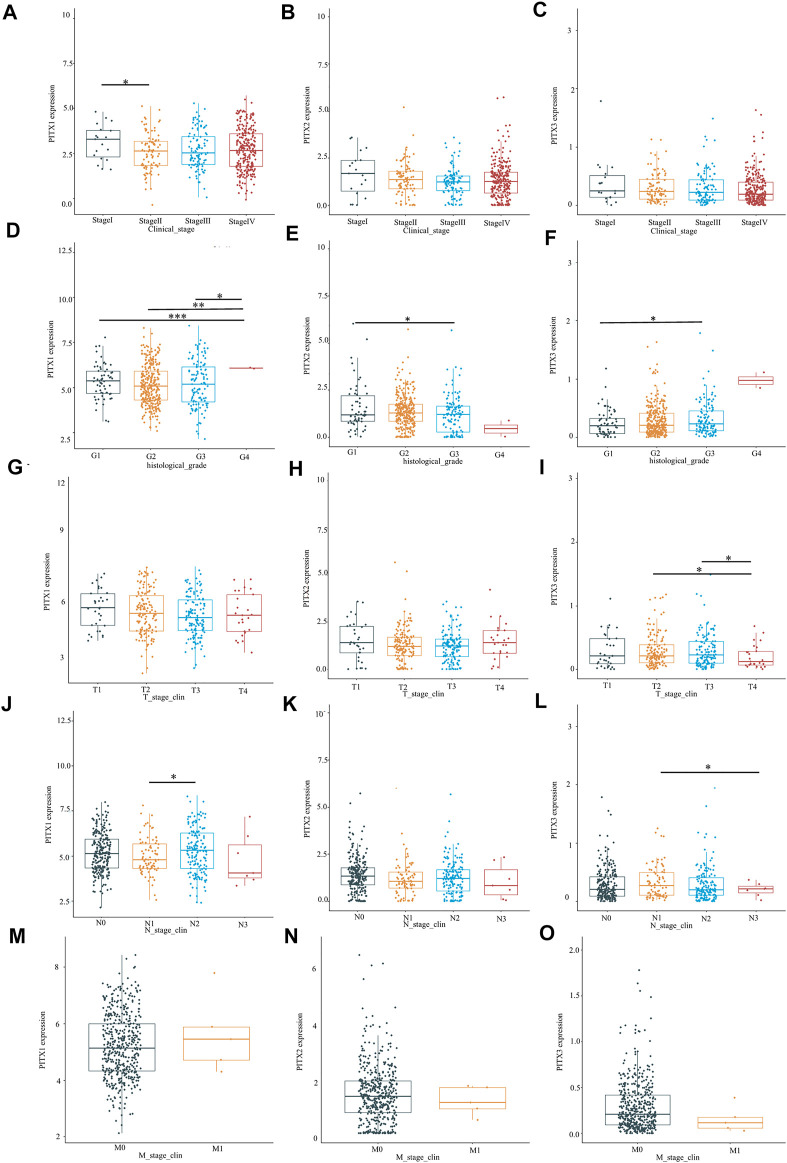
Relationship between PITX genes and clinicopathological parameters in patients with HNSC. The expression of *PITX1*, *PITX2*, and *PITX3* markedly correlated with **(A–C)** clinical stage, **(D–F)** histologic grade, **(G–I)** T stage, **(J–L)** N stage, and **(M–O)** M stage. *p* ≥ 0.05; *, *p* < 0.05; **, *p* < 0.01; ***, *p* < 0.001.

The clinicopathological characteristics of 502 patients with HNSC are summarized in Table 1. Roughly half of the HNSC patients were over 60 years old (51.1%) and had stage IV cancer (55.7%). The *PITX1* expression was significantly related to the clinical stage (stage I vs. stage II, *p* < 0.05, Figure 2A), histologic grade (G1 vs. G4, G2 vs. G4, G3 vs. G4, *p* < 0.05, Figure 2D), and N stage (N1 vs. N2, *p* < 0.05, Figure 2J) of HNSC. However, no relationship was detected between *PITX1* expression and HNSC T stage (*p >* 0.05, Figure 2G) or M stage (*p >* 0.05, Figure 2M). The *PITX2* expression was only significantly related to the histologic grade of HNSC (G1 vs. G3, *p* < 0.05, Figure 2E). No correlations were detected between *PITX2* expression and HNSC clinical stage (*p >* 0.05, Figure 2B), T stage (*p >* 0.05, Figure 2H), N stage (*p >* 0.05, Figure 2K), or M stage (*p >* 0.05, Figure 2N). The *PITX3* mRNA expression was significantly related to the histologic grade (G1 vs. G3, *p* < 0.05, Figure 2F), T stage (T2 vs. T4, T3 vs. T4, *p* < 0.05, Figure 2I), and N stage (N1 vs. N3, *p* < 0.05, Figure 2L) of HNSC. Meanwhile, no correlations were detected between *PITX3* expression and the HNSC clinical stage (*p >* 0.05, Figure 2C) or M stage (*p >* 0.05, Figure 2O).

**TABLE 1:** Clinical characteristics of the HNSC patients (from TCGA).

Characteristic	Levels	Overall
N		502
T stage, n (%)	T1	33 (6.8%)
	T2	144 (29.6%)
	T3	131 (26.9%)
	T4	179 (36.8%)
N stage, n (%)	N0	239 (49.8%)
	N1	80 (16.7%)
	N2	154 (32.1%)
	N3	7 (1.5%)
M stage, n (%)	M0	472 (99%)
	M1	5 (1%)
Clinical stage, n (%)	Stage I	19 (3.9%)
	Stage II	95 (19.5%)
	Stage III	102 (20.9%)
	Stage IV	272 (55.7%)
Gender, n (%)	Female	134 (26.7%)
	Male	368 (73.3%)
Race, n (%)	Asian	10 (2.1%)
	Black or African American	47 (9.7%)
	White	428 (88.2%)
Age, n (%)	<=60	245 (48.9%)
	>60	256 (51.1%)
Histologic grade, n (%)	G1	62 (12.8%)
	G2	300 (62.1%)
	G3	119 (24.6%)
	G4	2 (0.4%)
Smoker, n (%)	No	111 (22.6%)
	Yes	381 (77.4%)
Lymphovascular invasion, n (%)	No	219 (64.2%)
	Yes	122 (35.8%)
Lymph node neck dissection, n (%)	No	90 (18%)
	Yes	409 (82%)
OS event, n (%)	Alive	284 (56.6%)
	Dead	218 (43.4%)
DSS event, n (%)	Alive	347 (72.7%)
	Dead	130 (27.3%)
PFI event, n (%)	Alive	308 (61.4%)
	Dead	194 (38.6%)

DSS, disease-specific survival; PFI, progression-free interval.

### Prognostic value of *PITX* mRNA expression in HNSC patients

**FIGURE 3:**
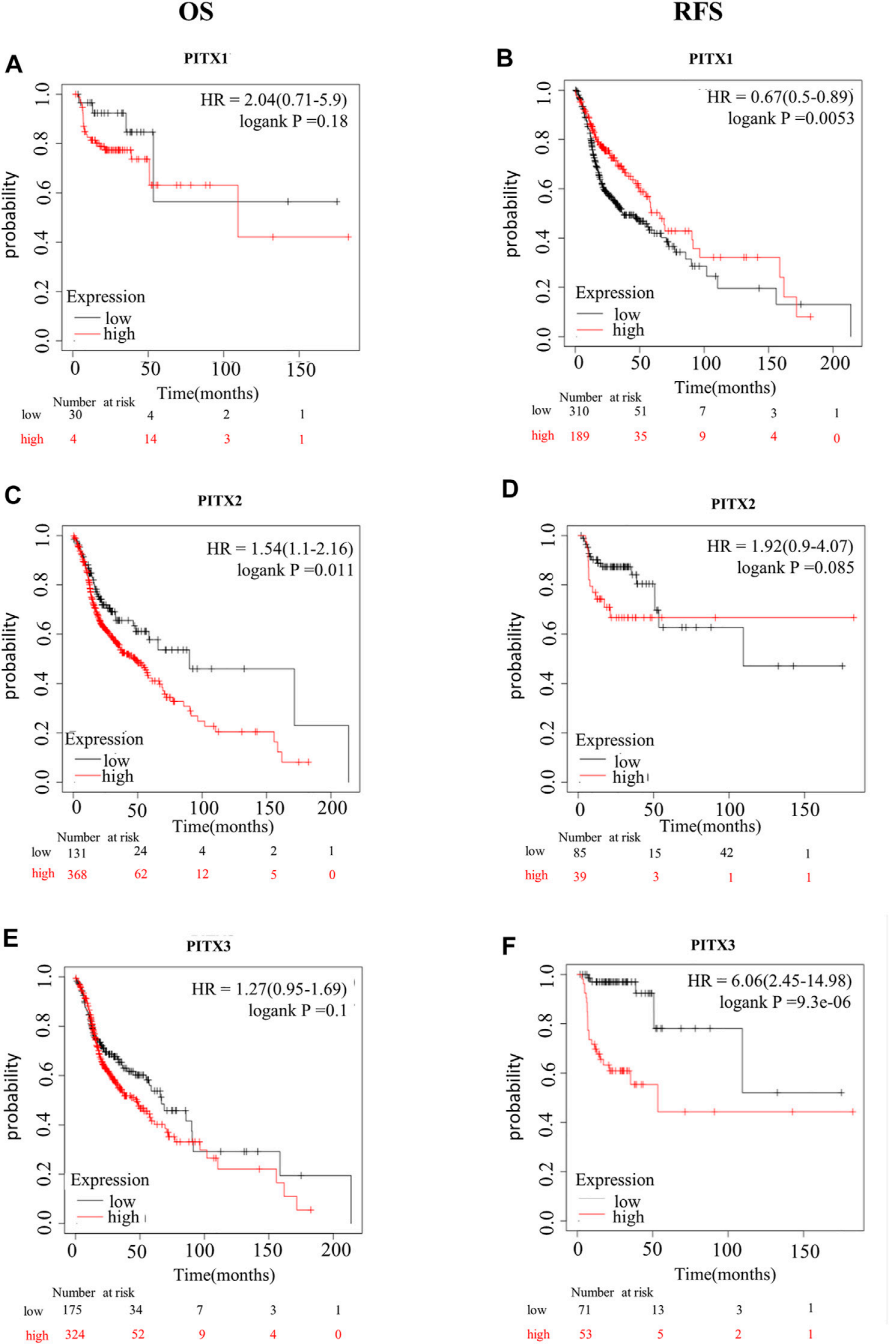
Kaplan-Meier survival curves for HNSC prognostic PITX genes. Kaplan-Meier survival curves for *PITX1* showing **(A)** OS and **(B)** RFS; Kaplan-Meier survival curves for *PITX2* showing **(C)** OS and **(D)** RFS; Kaplan-Meier survival curves for *PITX3* showing **(E)** OS and **(F)** RFS.

Next, we assessed the prognostic value of differentially expressed *PITX* genes in the context of HNSC. The correlations between different *PITX* genes and clinical outcomes were analyzed using the Kaplan-Meier plotter (Figure 3). We found that mRNA levels of *PITX1* were significantly correlated with RFS but not with OS in all HNSC patients. HNSC patients with higher *PITX1* mRNA levels *PITX1* had better RFS [hazard ratio (HR) = 0.67 (0.5–0.89), *p* = 0.0053]. *PITX2* expression was, however, significantly correlated with OS [HR = 1.54 (1.1–2.16), *p* = 0.011] but not with RFS. The expression of *PITX3* was associated with RFS but not with OS. In contrast to *PITX1*, higher *PITX3* mRNA levels were associated with poorer RFS in patients with HNSC [HR = 6.06 (2.45–14.98), *p* = 9.3e−06].

Next, univariate Cox regression analysis revealed that *PITX1* and *PITX2* co-expression was significantly correlated with OS. Subsequently, multivariate Cox regression analysis indicated that *PITX1* (HR = 0.832, *p* < 0.05) and *PITX2* (HR = 1.149, *p* < 0.05) exhibited independent prognostic values for HNSC (Table 2).

**TABLE 2:** Univariate and multivariate Cox analysis of *PITX* gene expression for determining HNSC prognosis.

Gene name	Total (N)	Univariate analysis		Multivariate analysis	
		Hazard ratio (95% CI)	*p-*value	Hazard ratio (95% CI)	*p*-value
*PITX1*	501	0.846 (0.750–0.954)	**0.006**	0.832 (0.736–0.940)	**0.003**
*PITX2*	501	1.118 (0.989–1.263)	0.073	1.149 (1.012–1.304)	**0.032**
*PITX3*	501	1.216 (0.753–1.962)	0.424		

Bold value means statistically significant.

### PITX1 and PITX2 PPI network and enrichment analysis

**FIGURE 4:**
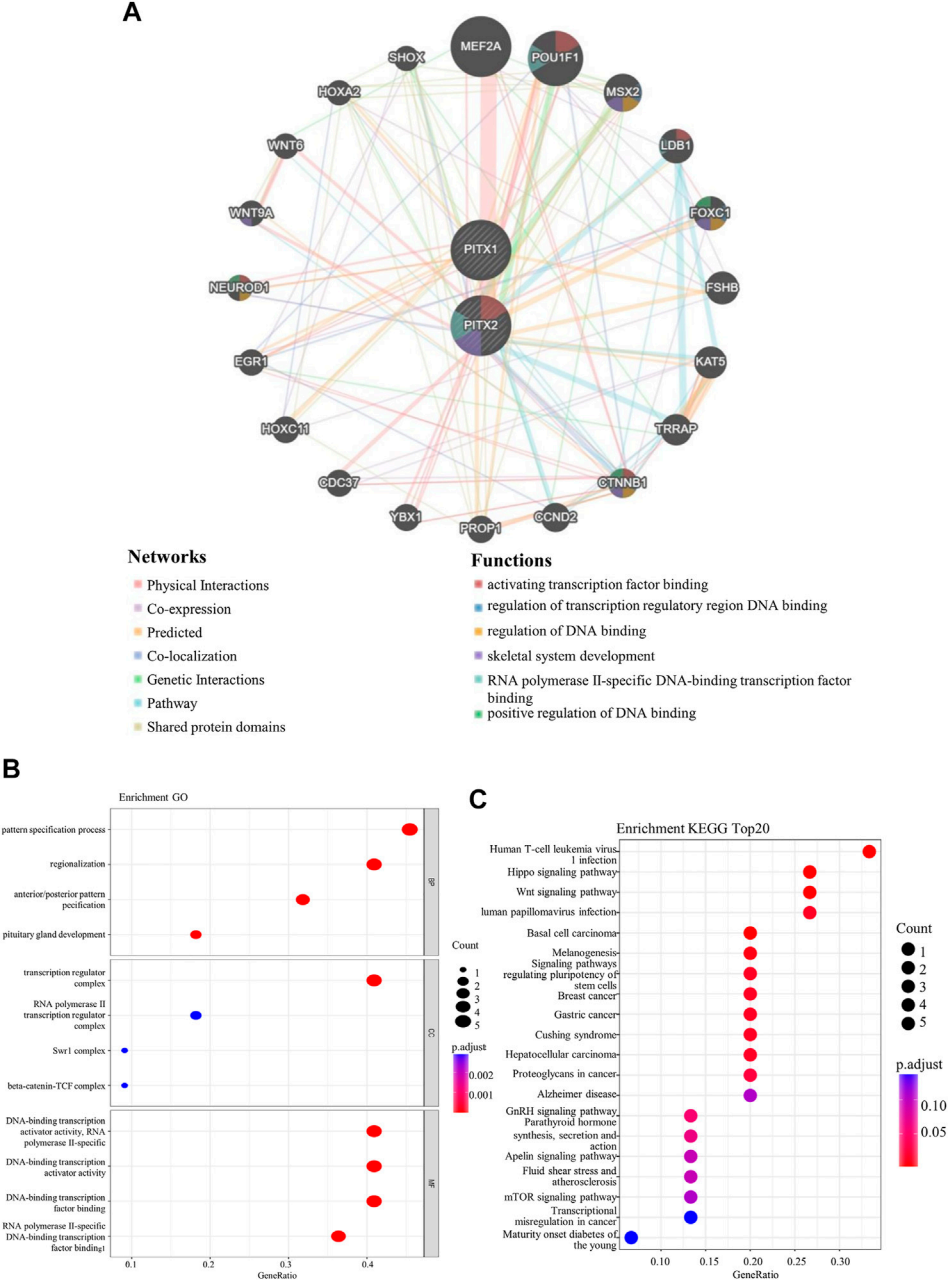
The network and functional enrichment analysis of PITX1 and PITX2. **(A)** The protein-protein interaction (PPI) network for *PITX1* and *PITX2* (generated using GeneMANIA). The functions of *PITX1*, *PITX2*, and co-expressed genes were predicted by performing **(B)** GO and **(C)** KEGG analysis.

Using GeneMANIA, we conducted a PPI network analysis of PITX1 and PITX2 to explore the potential interactions. We found that the *PITX* genes interacted with genes involved in the binding of activating transcription factors, the regulation of transcription regulatory region DNA binding, the regulation of DNA binding, skeletal system development, RNA polymerase II-specific DNA-binding transcription factor binding, and positive regulation of DNA binding (Figure 4A).

The functions of *PITX1*, *PITX2*, and the associated co-expressed genes were predicted by performing GO and KEGG analysis. GO enrichment analysis predicts the functions of target host genes based on biological processes, cell components, and molecular functions. We found that the pattern specification process, regionalization, anterior/posterior pattern specification, transcription regulator complex, DNA-binding transcription activator activity RNA polymerase II-specific, DNA-binding transcription activator activity, DNA-binding transcription factor binding, and RNA polymerase II-specific DNA-binding transcription factor binding were markedly associated with *PITX1* and *PITX2* alterations in HNSC (Figure 4B and Supplementary Table S1). The corresponding genes are known to be associated with DNA-binding transcription.

KEGG analysis can define the pathways related to the functions of altered *PITX1* and *PITX2* genes, and the associated co-expressed genes. Twelve pathways related to the functions of *PITX1* and *PITX2* alterations in HNSC were found (Figure 4C and Supplementary Table S2), including Human T-cell leukemia virus1 infection, Hippo signaling pathway, Wnt signaling pathway, Human papillomavirus infection, and so on.

### DNA methylation in promoter regions of *PITX1* and *PITX2*

**FIGURE 5:**
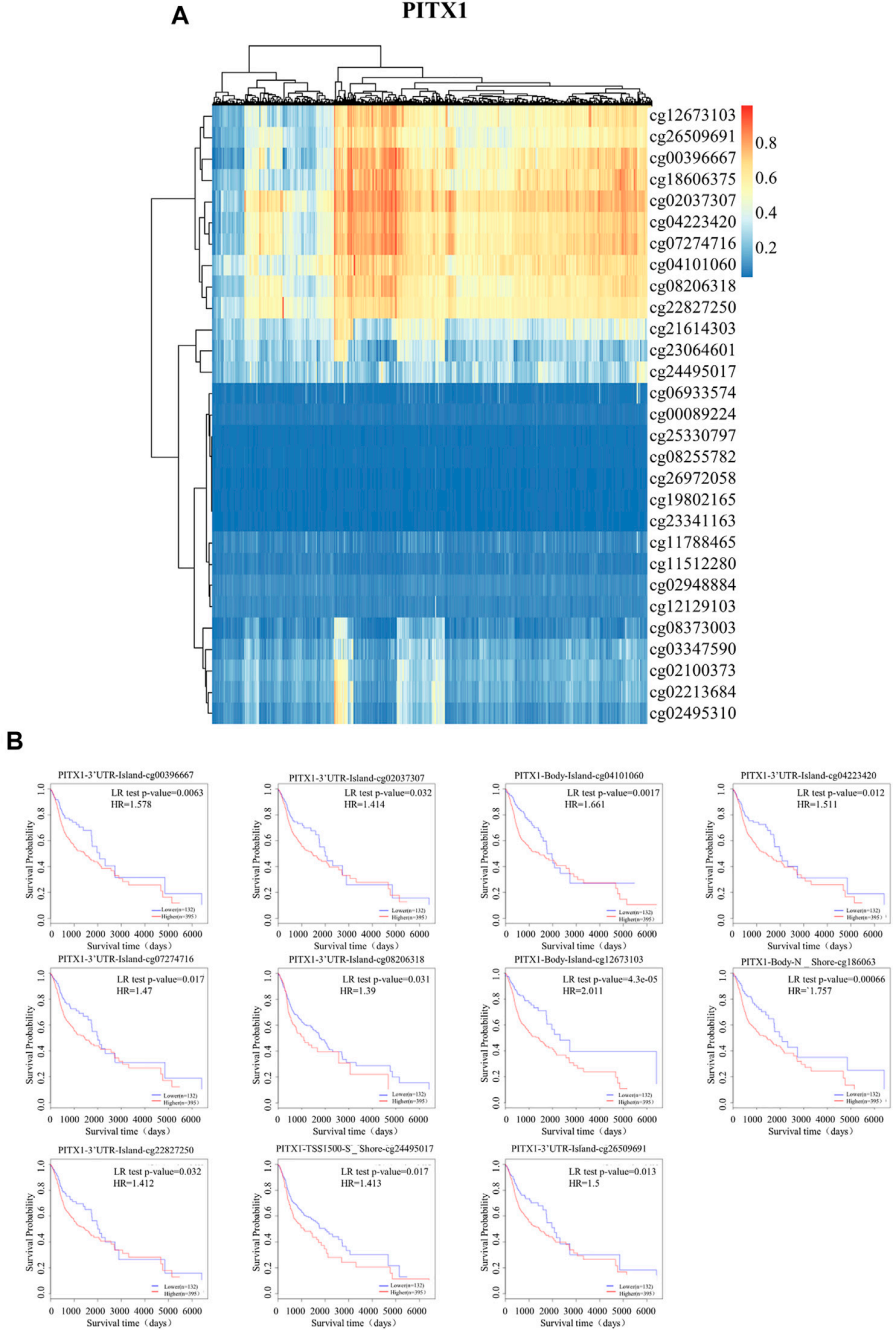
DNA methylation of PITX1 and HNSC prognosis. **(A)** The DNA methylation of *PITX1* was determined using MethSurv; **(B)** The prognostic values of CpGs in *PITX1*, based on Kaplan-Meier survival analysis. Red to blue: high expression to low expression.

**FIGURE 6:**
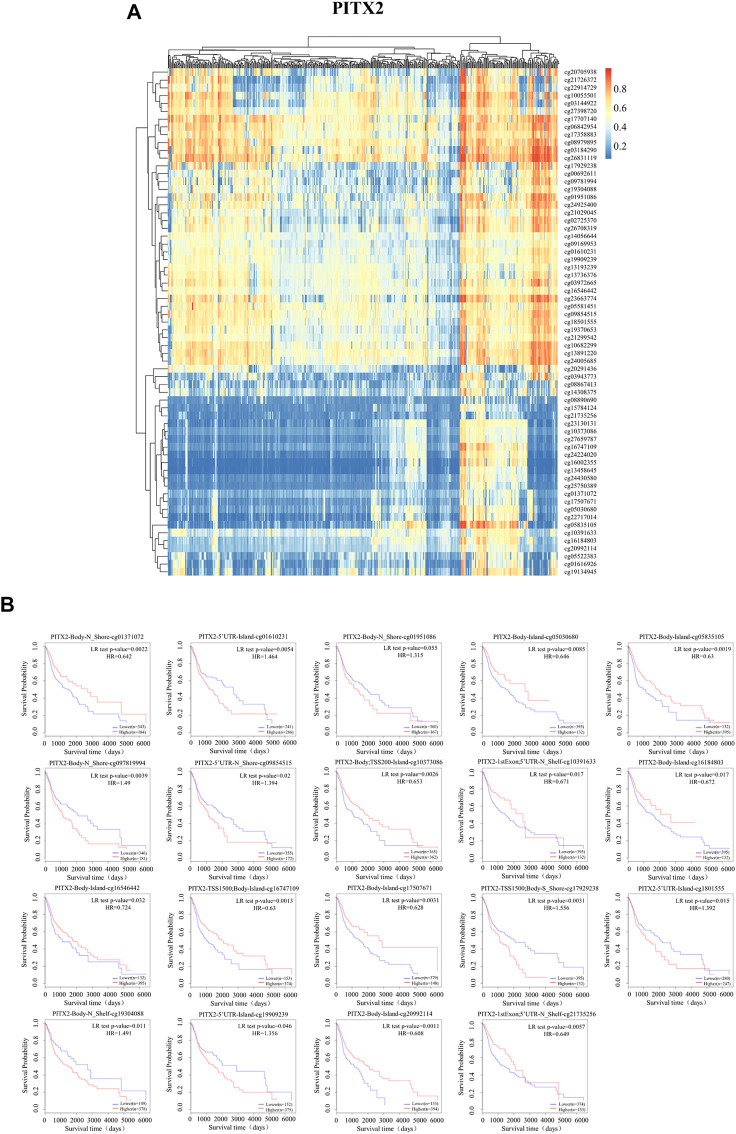
DNA methylation of PITX2. **(A)** The DNA methylation of *PITX2* was determined using MethSurv; **(B)** The prognostic values of CpGs in *PITX2*, based on Kaplan-Meier survival analysis. Red to blue: high expression to low expression.

The results of GO and KEGG analysis showed that *PITX1*, *PITX2*, and the associated genes were mainly enriched for DNA transcription-related functions. Therefore, the promoter methylation level of *PITX1* (Figure 5) and *PITX2* (Figure 6) were analyzed by MethSurv; the prognostic value of methylation at each CpG site was assessed individually. We found that cg02037307 of *PITX1* had the highest level of DNA methylation (Figure 5A). In addition, among the 29 CpGs in the *PITX1* promoter region, 11 had significant prognostic value for patients with HNSC, based on Kaplan-Meier survival analysis (Figure 5B); data for the remaining 18 CpGs are shown in Supplementary Figure S1. *PITX2* has 65 CpGs in the promoter region, of which cg26831119 had the highest level of methylation (Figure 6A). Nineteen of these CpGs had a significant prognostic value for HNSC, according to the Kaplan-Meier survival analysis (Figure 6B); data for the remaining 46 CpGs are shown in Supplementary Figure S2.

### The expression and methylation levels of *PITX1* and *PITX2* correlate with immune infiltration in HNSC

**FIGURE 7:**
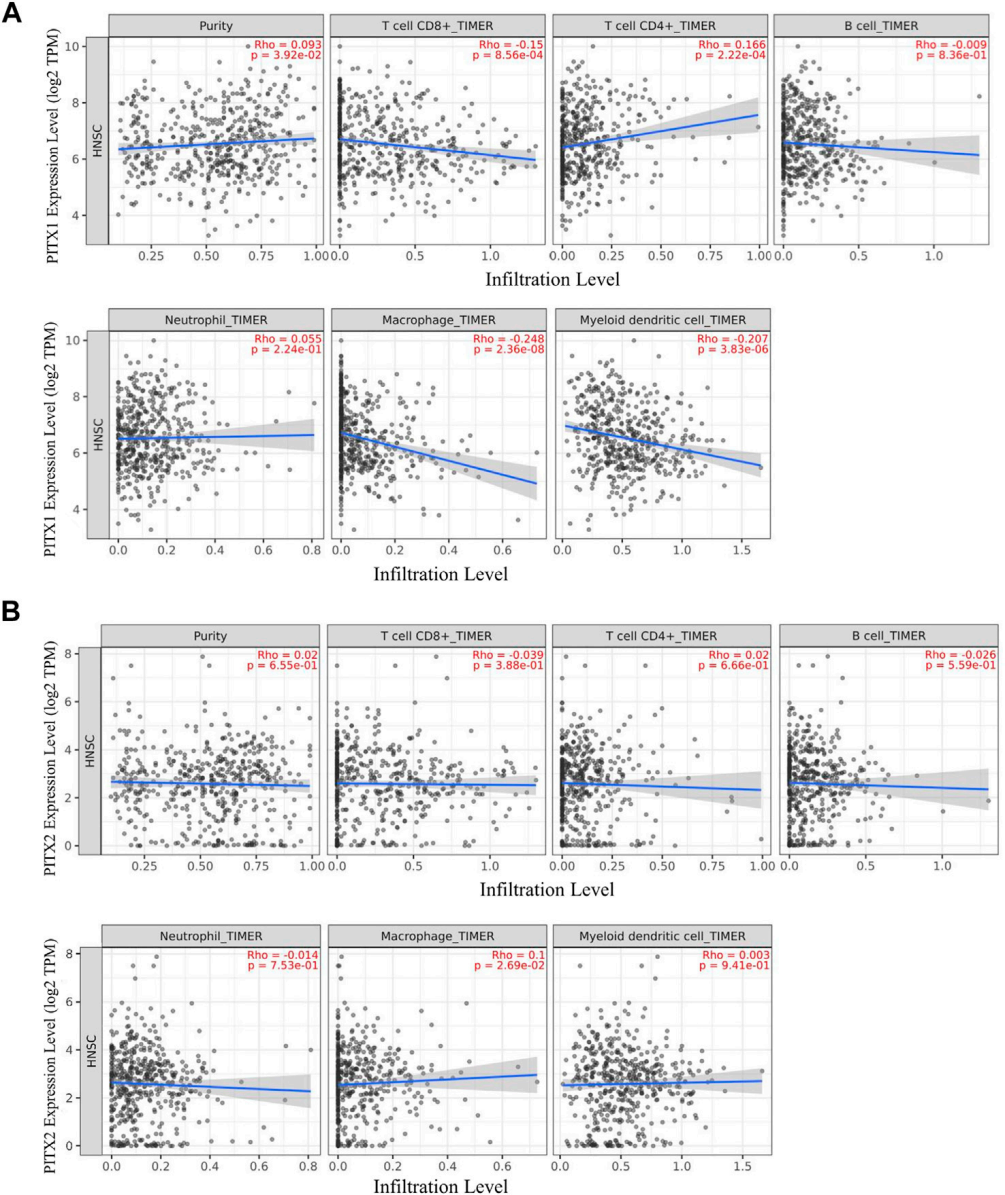
TIMER analysis of correlations between PITX1 and PITX2 expression and immune cell infiltration in HNSC. **(A)** Correlations between *PITX1* expression and immune cell infiltration were evaluated using TIMER. **(B)** Correlation between *PITX2* expression and immune cell infiltration levels using TIMER.

TIMER 2.0 is a tool for analyzing the associated between specific genes and immune infiltration. We used TIMER 2.0 to investigate the correlation between the expression of *PITX1* and *PITX2* and the infiltration levels of immune cells in HNSC. The expression of *PITX1* was significantly correlation with tumor-infiltrating CD8^+^ T cells (Rho = −0.15, *p* = 8.56e−04), CD4^+^ T cells (Rho = 0.166, *p* = 2.22e−04), macrophages (Rho = −0.248, *p* = 2.36e−08), and DCs (Rho = −0.207, *p* = 3.83e−06) (Figure 7A). Additionally, a correlation between *PITX2* expression and macrophage infiltrations (Rho = 0.1, *p* = 2.69e−02) was observed (Figure 7B).

**FIGURE 8:**
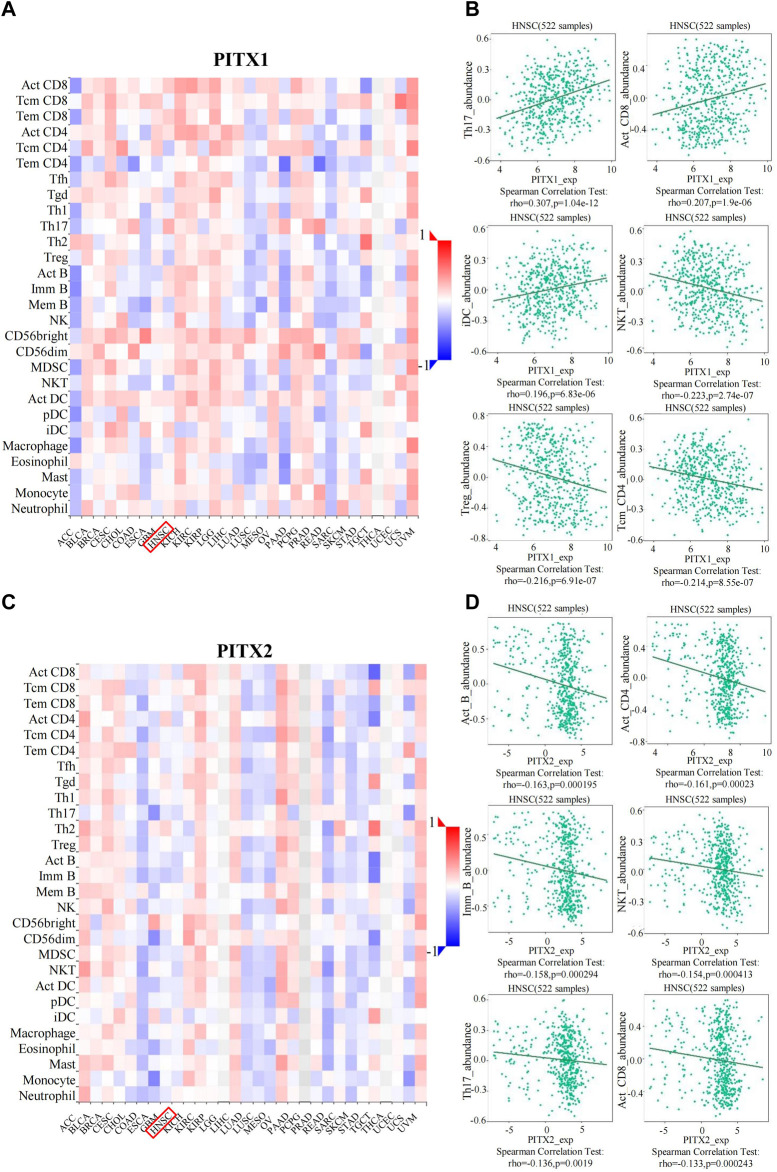
TISIDB analysis of correlations between PITX1 and PITX2 expression and immune cell infiltration across human cancers. **(A)** The relationship between *PITX1* expression and 28 types of TILs across various human cancers. **(B)** The top six TILs displaying the strongest Spearman’s correlation with *PITX1* expression in HNSC. **(C)** The relationship between *PITX2* expression and 28 types of TILs across various human cancers. **(D)** The top six TILs displaying the strongest Spearman’s correlation with *PITX2* expression in HNSC. Act_CD8, activated CD8^+^ T cells; Tcm_CD8, central memory CD8^+^ T cells; Tem_CD8, effector memory CD8^+^ T cells; Act_CD4, activated CD4^+^ T cells; Tcm_CD4, central memory CD4^+^ T cells; Tem_CD4, effector memory CD4^+^ T cells; Tfh, T follicular helper cells; Tgd, gamma delta T cells; Th1, type 1 T helper cells; Th17, type 17 T helper cells; Th2, type 2 T helper cells; Treg, regulatory T cells; Act B, activated B cells; Imm_B, immature B cells; Mem_B, memory B cells; NK, natural killer cells; CD56^bright^, CD56^bright^ NK cells; CD56^dim^, CD56^dim^ NK cells; MDSC, myeloid derived suppressor cells; NKT, natural killer T cells; Act DC, activated dendritic cells; pDC, plasmacytoid DCs; iDC, immature DCs; Mast, mast cell.

TILs can independently be used to predict sentinel lymph node positivity and cancer prognosis (Warde-Farley et al., 2010). Therefore, we used TISIDB to further explore the relationship between TIL numbers and the expression or methylation of *PITX1* and *PITX2*. We found that the expression of *PITX1* was associated with 16 immune cell subtypes in HNSC (Figure 8A and Table 3). The strongest associations with *PITX1* expression involved T helper (Th)17 cells (*r* = 0.307, *p* = 1.04E−12), activated CD8^+^ T cells (Act_CD8, *r* = 0.207, *p* = 1.9E−06), immature (i) DCs (r = 0.196, *p* = 6.83E-06), natural killer T (NKT) cells (*r* = −0.223, *p* = 2.74E−07), regulatory T cells (Tregs) (*r* = −0.216, *p* = 6.91E−07), and central memory CD4^+^ T cells (Tcm_CD4, *r* = −0.214, *p* = 8.55E−07) (Figure 8B). The expression of *PITX2* was associated with nine immune cell subtypes in HNSC (Figure 8C and Table 3). Among these, activated B cells (Act_B, *r* = −0.163, *p* = 0.000195), activated CD4^+^ T cells (Act_CD4, *r* = −0.161, *p* = 0.00023), immature B cells (Imm_B, *r* = −0.158, *p* = 0.000294), NKT cells (*r* = −0.154, *p* = 0.000413), Th17 cells (*r* = −0.136, *p* = 0.0019), and Act_CD8 (*r* = −0.133, *p* = 0.00243) displayed the strongest correlations with *PITX2* expression (Figure 8D).

**TABLE 3:** The expression and methylation levels of *PITX1* and *PITX2* correlate with ITLs.

	*PITX1* expression		*PITX2* expression		*PITX1* methylation		*PITX2* methylation	
	r	p	r	p	r	p	r	p
Activated CD8^+^ T cell (act_CD8)	0.207	**1.9e−06**	−0.133	**0.00243**	0.346	**5.28e−16**	0.357	**<2.20e−16**
Central memory CD8^+^ T cell (Tcm_CD8)	−0.06	0.174	0.076	0.0818	0.375	**<2.20e−16**	−0.065	0.139
Effector memory CD8^+^ T cell (Tem_CD8)	0.195	**7.22e−06**	−0.078	0.0744	0.365	**9.4e−19**	0.343	**1.02e−15**
Activated CD4^+^ T cell (Act CD4)	0.117	**0.00742**	−0.161	**0.00023**	0.12	**0.00629**	0.368	**<2.20e−16**
Central memory CD4^+^ T cell (Tcm_CD4)	−0.214	**8.55e−07**	−0.051	0.248	0.393	**<2.20e−16**	−0.062	0.16
Effector memory CD4^+^ T cell (Tem_CD4)	−0.029	0.503	−0.028	0.523	0.2	**4.26e−06**	0.218	**5.14e−07**
T follicular helper cell (Tfh)	−0.047	0.283	−0.033	0.446	0.478	**<2.20e−16**	0.203	**3.22e−06**
Gamma delta T cell (Tgd)	−0.173	**7.26e−05**	−0.05	0.255	0.442	**<2.20e−16**	0.006	0.885
Type 1 T helper cell (Th1)	−0.068	0.123	−0.042	0.341	0.437	**<2.20e−16**	0.27	**4.4e−10**
Type 17 T helper cell (Th17)	0.307	**1.04e−12**	−0.136	**0.0019**	0.091	**0.0375**	0.371	**<2.20e−16**
Type 2 T helper cell (Th2)	−0.077	0.0789	−0.034	0.434	0.2	**4.52e−06**	0.146	**0.000843**
Regulatory T cell (Treg)	−0.216	**6.91e−07**	−0.082	0.0617	0.475	**<2.20e−16**	0.134	**0.00225**
Activated B cell (act B)	0.148	**0.000717**	−0.163	**0.000195**	0.259	**<2.20e−16**	0.477	**<2.20e−16**
Immature B cell (Imm_B)	0.118	**0.00718**	−0.158	**0.000294**	0.371	**<2.20e−16**	0.431	**<2.20e−16**
Memory B cell (Mem_B)	−0.178	**4.37e−05**	0.019	0.673	0.176	**5.36e−05**	0.187	**1.76e−05**
Natural killer cell (NK)	−0.069	0.113	−0.049	0.26	0.318	**1.25e−13**	0.225	**2.27e−07**
CD56^bright^ natural killer cell (CD56^bright^)	0.102	**0.0193**	−0.005	0.904	0.274	**2.28e−10**	−0.071	0.107
CD56^dim^ natural killer cell (CD56^dim^)	0.029	0.51	0.012	0.793	0.113	**0.00974**	0.007	0.877
Myeloid derived suppressor cell (MDSC)	0.082	0.0626	−0.098	**0.025**	0.458	**<2.20e−16**	0.273	**2.83e−10**
Natural killer T cell (NKT)	−0.223	**2.74e−07**	−0.154	**0.000413**	0.391	**<2.20e−16**	0.184	**2.38e−05**
Activated dendritic cell (Act_DC)	0.108	**0.0135**	−0.08	0.0664	0.336	**3.66e−15**	0.223	**2.68e−07**
Plasmacytoid dendritic cell (pDC)	−0.041	0.353	−0.092	**0.0363**	0.276	**1.72e−10**	0.091	**0.0376**
Immature dendritic cell (iDC)	0.196	**6.83e−06**	0.072	0.101	0.158	**0.000293**	0.126	**0.00383**
Macrophage (macrophage)	−0.1	**0.023**	−0.041	0.35	0.425	**<2.20e−16**	0.188	**1.59e−05**
Eosinophil (eosinophil)	0.041	**<2.20e−16**	−0.056	0.203	0.242	**2.39e−08**	0.249	**9.54e−09**
Mast cell (mast)	−0.028	0.525	−0.022	0.614	0.227	**1.37e−10**	0.186	**2e−05**
Monocyte (monocyte)	0.038	0.389	−0.11	**0.0118**	0.396	**<2.20e−16**	0.154	**0.000438**
Neutrophil (neutrophil)	0.091	**0.0371**	0.041	0.346	0.239	**3.41e−08**	−0.021	0.637

Bold value means statistically significant.

**FIGURE 9:**
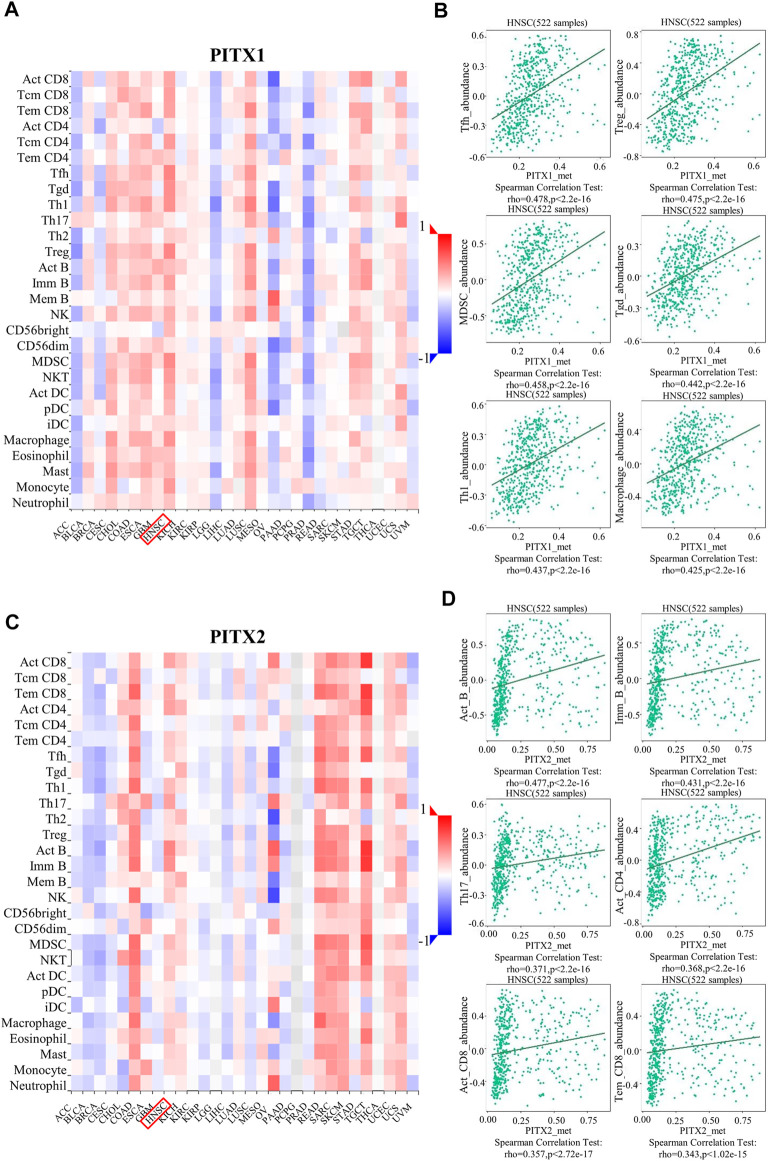
TISIDB analysis of correlations between PITX1 and PITX2 methylation and immune cells infiltration across human cancers. **(A)** The relationship between *PITX1* methylation and 28 types of TILs across various human cancers. **(B)** The top six TILs displaying the strongest Spearman’s correlation with *PITX1* methylation in HNSC. **(C)** The relationship between *PITX2* methylation and 28 types of TILs across various human cancers. **(D)** The top six TILs displaying the strongest Spearman’s correlation with *PITX2* methylation in HNSC. Act_CD8, activated CD8^+^ T cells; Tcm_CD8, central memory CD8^+^ T cells; Tem_CD8, effector memory CD8^+^ T cells; Act_CD4, activated CD4^+^ T cells; Tcm_CD4, central memory CD4^+^ T cells; Tem_CD4, effector memory CD4^+^ T cells; Tfh, T follicular helper cells; Tgd, gamma delta T cells; Th1, type 1 T helper cells; Th17, type 17 T helper cells; Th2, type 2 T helper cells; Treg, regulatory T cells; Act B, activated B cells; Imm_B, immature B cells; Mem_B, memory B cells; NK, natural killer cells; CD56^bright^, CD56^bright^ NK cells; CD56^dim^, CD56^dim^ NK cells; MDSC, myeloid derived suppressor cells; NKT, natural killer T cells; Act DC, activated dendritic cells; pDC, plasmacytoid DCs; iDC, immature DCs; Mast, mast cells.

In addition, the methylation level of *PITX1* and *PITX2* had a significant impact on the prognosis of HNSC. We analyzed the correlation between TILs and the methylation level of *PITX1* and *PITX2* using the TISIDB. Our results showed that the methylation level of *PITX1* was associated with 28 types of TILs in HNSC (Figure 9A and Table 3). Follicular helper T (Tfh) cells (*r* = 0.478, *p* < 2.2E−16), Tregs (*r* = 0.475, *p* < 2.2E−16), myeloid-derived suppressor cells (MDSCs) (*r* = 0.458, *p* < 2.2E−16), NKT cells (*r* = 0.442, *p* < 2.2E−16), Th1 cells (*r* = 0.437, *p* < 2.2E-16), and macrophages (*r* = 0.425, *p* < 2.2E-16) were most strongly correlated (*r* > 0.3) with *PITX1* methylation (Figure 9B). *PITX2* methylation was associated with 22 immune cell subtypes in HNSC (Figure 9C and Table 3). Among these TILs, Act_B (*r* = 0.477, *p* < 2.2E−16), Imm_B (*r* = 0.431, *p* < 2E−16), Th17 cells (*r* = 0.371, *p* < 2.2E−16), Act_CD4 (*r* = 0.368, *p* < 2.2E-16), Act_CD8 (*r* = 0.357, *p* < 2.2E−16), and effector memory CD8^+^ T cells (Tem_CD8, *r* = 0.343, *p* = 1.02E−15) were most strongly correlated with *PITX2* methylation (Figure 9D). These results imply that the methylation level of *PITX1* and *PITX2* could serve as a regulator of immune cell infiltration into HNSC tumors.

## Discussion

The dysregulation of the *PITX* gene family has been reported in various types of cancer (Tran and Kioussi, 2021). Although the role of *PITX* genes in the initiation and prognosis of certain cancers has been partially demonstrated (Zhang et al., 2021), further bioinformatic analysis of their involvement in HNSC need to be performed. To the best of our knowledge, this study was the first attempt to investigate the oncological characteristics and prognostic value of different members of the *PITX* gene family in HNSC.

In the present study, we demonstrated that the expression of the *PITX* gene family in HNSC is related to some clinicopathological features, including pathologic types and histologic grade (according to TCGA). We also found that the expression of *PITX* genes correlated with the prognosis of patients with HNSC. OS and RFS are commonly used as primary end points to assess prognostic value of a particular marker (Jabbour et al., 2018). Our bioinformatic analysis showed that *PITX1* expression was decreased in patients with HNSC, which correlated with relatively poor RFS. Moreover, the Student’s *t*-test values indicated that *PITX1* was significantly associated with tumor stage, histologic grade, and N stage, which are related to cancer progression. Univariate/multivariate Cox regression analysis results also indicated that the expression of *PITX1* could be an independent prognostic factor for HNSC. Collectively, these results revealed that *PITX1* may perform well as a prognostic predictor for HNSC.

*PITX1* acts as a tumor suppressor gene in various human cancers (Kolfschoten et al., 2005). It was reported that the downregulation of *PITX1* expression might contribute to the progression of cutaneous malignant melanoma by promoting cell proliferation (Osaki et al., 2013). In lung cancer, *PITX1* expression was also decreased, with 62% of lung cancer patients displaying no evidence of *PITX1* expression. Moreover, the lack of *PITX1* mRNA expression was associated with a higher tumor grade (Chen et al., 2007). The low *PITX1* expression in HNSC may therefore be correlated with a worse prognosis. In our study, the expression of *PITX2* in patients with HNSC was lower than that in normal tissues; however, its expression was not correlated with tumor histologic stage, T stage, N stage, M stage, or RFS. In addition, *PITX3* was proven to be associated with HNSC and serve as an independent prognostic biomarker (Sailer et al., 2017b). Consistently, we found that *PITX3* may be positively correlated with the occurrence and development of HNSC. The higher expression of *PITX3* in HNSC was associated with higher G and T stages and poorer RFS (Holmes et al., 2016). The high expression of *PITX3* in HNSC may therefore also lead to poor prognosis. PPI network and functional enrichment analysis showed that the high expression of PITX1 and PITX2 was mainly associated with DNA binding, regulation and transcription, transcription factor activation, and RNA polymerase II-specific processes. Dysregulation or mutation of DNA-binding proteins has been implicated in the development and progression of various types of diseases, including cancer (Shiroma et al., 2020). We also found that PITX1 and PITX2 co-expression was strongly associated with the myocyte enhancer factor 2A (MEF2A), while PITX2 expression alone was closely functionally related to pituitary-specific positive transcription factor 1 (POU1F1). A promoter- and cell-specific functional interaction between PITX2 and MEF2A was previously reported, which is involved in the regulation of oral epithelial cells (Toro et al., 2004). MEF2A may also promote the transcriptional activity of other factors, thus promoting stem-like properties of oral squamous cell carcinoma and playing an important role in the development of HNSC (Wang et al., 2022). Moreover, PITX2 plays a crucial role in embryogenesis, ontogenesis, growth, and development via the Wnt/beta-catenin and POU1F1 pathways (Zhang et al., 2018).

DNA methylation is the most common epigenetic marker that usually acts as a transcriptional repressor and contributes to tumor progression (Meng et al., 2015; Zhou et al., 2020). DNA methylation can directly affect the interaction between DNA-binding proteins with their cognate sites (Zhu et al., 2016). It has been reported that DNA methylation plays an important role in the etiology, pathogenesis, and prognosis of HNSC (Zhou et al., 2018). Thus, we were interested in investigating the interaction between DNA methylation and DNA-binding proteins in the context of HNSC. Our MethSurv database analysis revealed that *PITX1* and *PITX2* expression was strongly correlated with DNA methylation. Our study found that the expression of *PITX1* and *PITX2* was significantly lower in HNSC tumors, compared to normal tissues, which was related to *PITX1* and *PITX2* hypermethylation. A recent bioinformatics analysis showed that high *PITX1* expression was related to DNA methylation and poor prognosis in lung adenocarcinoma (Song et al., 2018). The DNA methylation level of *PITX2* was also associated with the risk of occurrence and progression of lung cancer (Dietrich et al., 2012). *PITX2* DNA methylation was proven to serve as a prognostic biomarker for patients with HNSC (Sailer et al., 2017a).

Cancers are inextricably linked with the immune response. The immune system acts as an external tumor suppressor but conversely can also promote cancer occurrence, development, and progression (Vesely et al., 2011). We therefore explored the potential relationship between *PITX* genes and immunity, focusing on the tumor microenvironment, tumor immune infiltration, and immune cell DNA methylation. As for tumor immune infiltration, *PITX1* and *PITX2* expression was positively correlated with the infiltration of Th17 cells and Act_CD8^+^ T cells, but negatively correlated with NKT cell infiltration. A recent clinical trial has shown that tumor immune cell infiltration is correlated with the sensitivity to immunotherapy and HNSC prognosis (Zhang et al., 2020). In tumors of the oral cavity, outcomes appear to improve with an increase in tumoral or stromal CD8^+^ T cell numbers (Oguejiofor et al., 2015). Individuals with more genetic defects affecting NKT cells had a higher risk of developing cancer and these inherited defects were associated with tumor immune microenvironment subtypes, recruitment of TILs, and clinical outcomes (Xu et al., 2019). Besides, we found that the methylation level of *PITX1* and *PITX2* was associated with various TILs in HNSC. DNA methylation plays a key role in TIL differentiation and plasticity, which can be associated with favorable or poor prognoses in many types of cancer (Zeiner et al., 2018; Merino et al., 2019; Renaude et al., 2020). Therefore, we speculate that the correlations that we have observed may help reveal the mechanism by which *PITX1* and *PITX2* regulate the function of immune cells in HNSC.

## Data Availability

The original contributions presented in the study are included in the article/Supplementary Material, further inquiries can be directed to the corresponding author.
