# Supplementary material for: The expression and methylation of PITX genes is associated with the prognosis of head and neck squamous cell carcinoma
Source: Front Genet. 2022 Sep 20;13:982241. doi: 10.3389/fgene.2022.982241 (PMC9530742; doi:10.3389/fgene.2022.982241)
Supplement: Supplementary file 1 [file DataSheet1.docx]

Supplementary Material

# Supplementary Figures and Tables

## Supplementary Figures

**
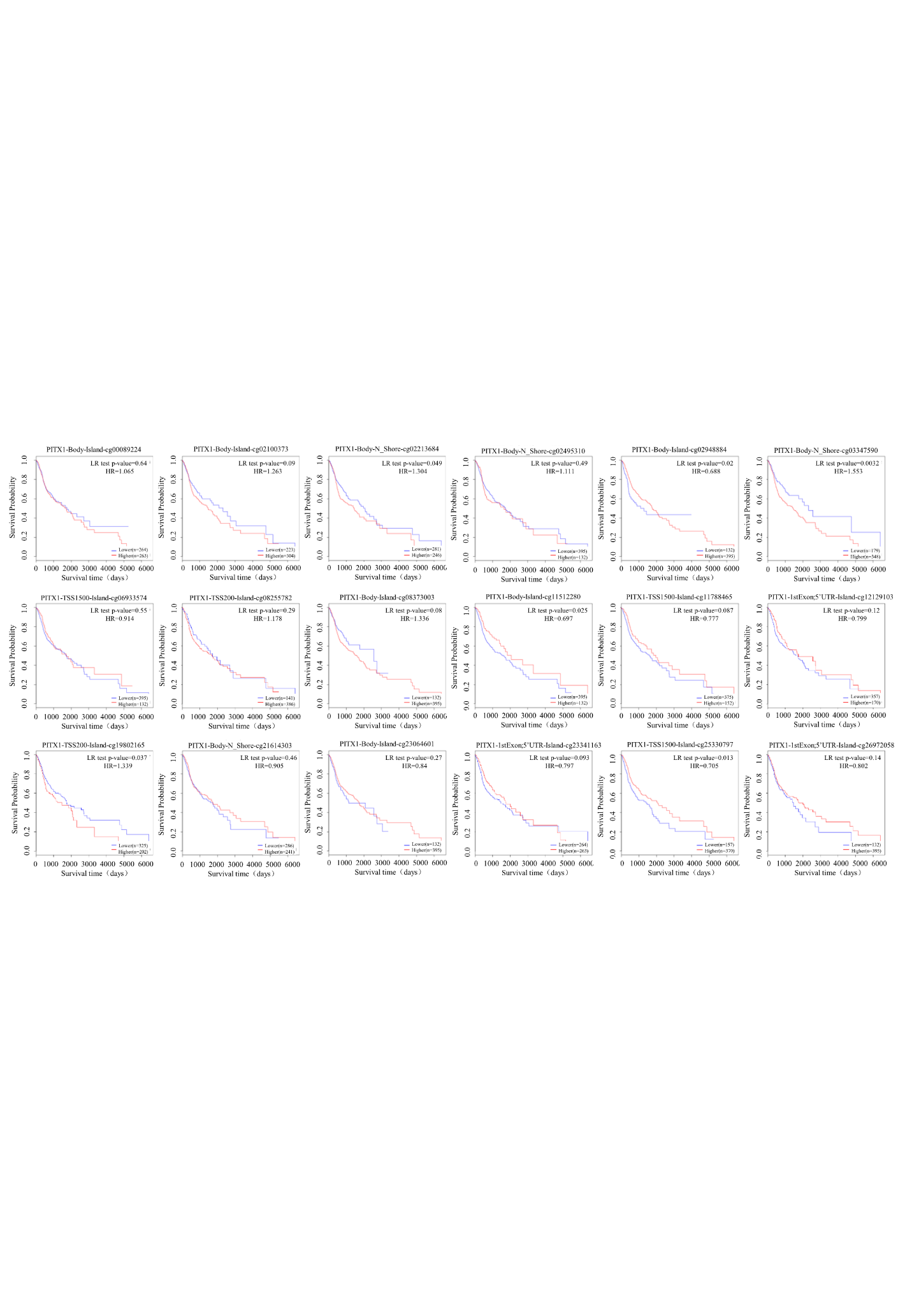
**

**Supplementary Figure 1.** The prognostic values of CpGs in PITX1 based on KM survival analysis.


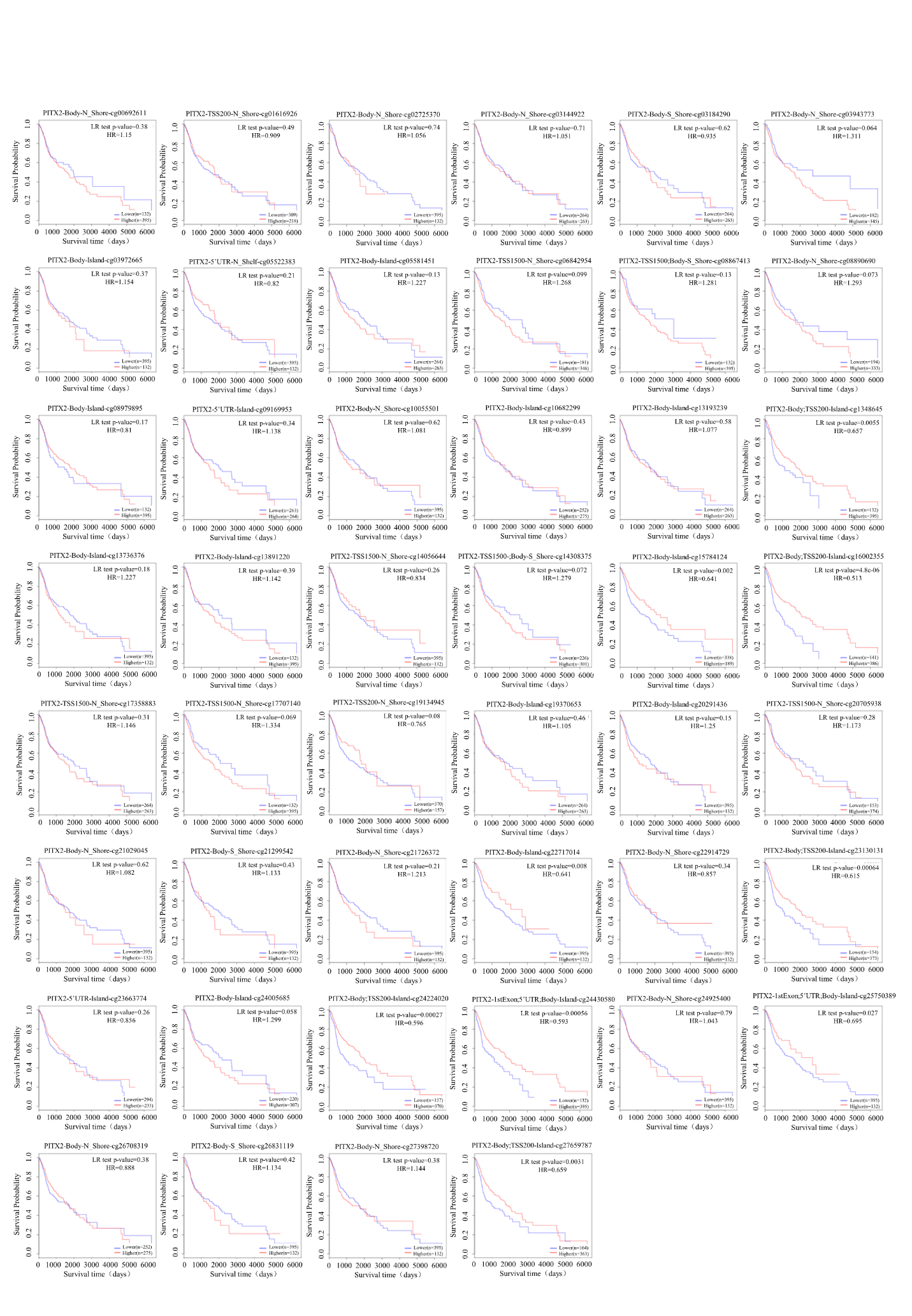


**Supplementary Figure 2.** The prognostic values of CpGs in PITX2 based on KM survival analysis.

## Supplementary Tables

| ONTOLOGY | ID | Description | GeneRatio | p.adjust | Count |
| --- | --- | --- | --- | --- | --- |
| BP | GO:0007389 | pattern specification process | 10/22 | 3.94E-08 | 10 |
| BP | GO:0003002 | regionalization | 9/22 | 5.75E-08 | 9 |
| BP | GO:0009952 | anterior/posterior pattern specification | 7/22 | 1.18E-06 | 7 |
| BP | GO:0021983 | pituitary gland development | 4/22 | 3.69E-05 | 4 |
| BP | GO:0042476 | odontogenesis | 5/22 | 5.35E-05 | 5 |
| BP | GO:2000677 | regulation of transcription regulatory region DNA binding | 4/22 | 5.35E-05 | 4 |
| BP | GO:0035270 | endocrine system development | 5/22 | 5.35E-05 | 5 |
| BP | GO:0048732 | gland development | 7/22 | 5.35E-05 | 7 |
| BP | GO:0045165 | cell fate commitment | 6/22 | 5.35E-05 | 6 |
| BP | GO:0021984 | adenohypophysis development | 3/22 | 5.35E-05 | 3 |
| BP | GO:0072498 | embryonic skeletal joint development | 3/22 | 6.08E-05 | 3 |
| BP | GO:0030902 | hindbrain development | 5/22 | 6.34E-05 | 5 |
| BP | GO:0048736 | appendage development | 5/22 | 0.00011 | 5 |
| BP | GO:0060173 | limb development | 5/22 | 0.00011 | 5 |
| BP | GO:0021536 | diencephalon development | 4/22 | 0.00011 | 4 |
| BP | GO:0043010 | camera-type eye development | 6/22 | 0.00011 | 6 |
| BP | GO:0003151 | outflow tract morphogenesis | 4/22 | 0.000132 | 4 |
| BP | GO:0008544 | epidermis development | 6/22 | 0.000149 | 6 |
| BP | GO:2000679 | positive regulation of transcription regulatory region DNA binding | 3/22 | 0.000155 | 3 |
| BP | GO:0042475 | odontogenesis of dentin-containing tooth | 4/22 | 0.000177 | 4 |
| BP | GO:0030900 | forebrain development | 6/22 | 0.000177 | 6 |
| BP | GO:0001654 | eye development | 6/22 | 0.000177 | 6 |
| BP | GO:0150063 | visual system development | 6/22 | 0.000178 | 6 |
| BP | GO:0009798 | axis specification | 4/22 | 0.000178 | 4 |
| BP | GO:0048880 | sensory system development | 6/22 | 0.000181 | 6 |
| BP | GO:0035116 | embryonic hindlimb morphogenesis | 3/22 | 0.000183 | 3 |
| BP | GO:0060537 | muscle tissue development | 6/22 | 0.000212 | 6 |
| BP | GO:0048705 | skeletal system morphogenesis | 5/22 | 0.000213 | 5 |
| BP | GO:0035137 | hindlimb morphogenesis | 3/22 | 0.000322 | 3 |
| BP | GO:0048568 | embryonic organ development | 6/22 | 0.000322 | 6 |
| BP | GO:0016055 | Wnt signaling pathway | 6/22 | 0.000322 | 6 |
| BP | GO:0030326 | embryonic limb morphogenesis | 4/22 | 0.000322 | 4 |
| BP | GO:0035113 | embryonic appendage morphogenesis | 4/22 | 0.000322 | 4 |
| BP | GO:0198738 | cell-cell signaling by wnt | 6/22 | 0.000322 | 6 |
| BP | GO:0061448 | connective tissue development | 5/22 | 0.000322 | 5 |
| BP | GO:0051101 | regulation of DNA binding | 4/22 | 0.000323 | 4 |
| BP | GO:0001894 | tissue homeostasis | 5/22 | 0.000371 | 5 |
| BP | GO:0048706 | embryonic skeletal system development | 4/22 | 0.000371 | 4 |
| BP | GO:0035107 | appendage morphogenesis | 4/22 | 0.000547 | 4 |
| BP | GO:0035108 | limb morphogenesis | 4/22 | 0.000547 | 4 |
| BP | GO:0001822 | kidney development | 5/22 | 0.000566 | 5 |
| BP | GO:0072006 | nephron development | 4/22 | 0.000566 | 4 |
| BP | GO:0072001 | renal system development | 5/22 | 0.000626 | 5 |
| BP | GO:0060070 | canonical Wnt signaling pathway | 5/22 | 0.000626 | 5 |
| BP | GO:0060249 | anatomical structure homeostasis | 5/22 | 0.000671 | 5 |
| BP | GO:0007517 | muscle organ development | 5/22 | 0.000817 | 5 |
| BP | GO:0043388 | positive regulation of DNA binding | 3/22 | 0.000886 | 3 |
| BP | GO:0042733 | embryonic digit morphogenesis | 3/22 | 0.000914 | 3 |
| BP | GO:0001655 | urogenital system development | 5/22 | 0.000986 | 5 |
| BP | GO:0071300 | cellular response to retinoic acid | 3/22 | 0.001353 | 3 |
| BP | GO:0051216 | cartilage development | 4/22 | 0.001428 | 4 |
| BP | GO:0060272 | embryonic skeletal joint morphogenesis | 2/22 | 0.001428 | 2 |
| BP | GO:0061303 | cornea development in camera-type eye | 2/22 | 0.001428 | 2 |
| BP | GO:1904888 | cranial skeletal system development | 3/22 | 0.001428 | 3 |
| BP | GO:0018205 | peptidyl-lysine modification | 5/22 | 0.001463 | 5 |
| BP | GO:0044848 | biological phase | 2/22 | 0.001631 | 2 |
| BP | GO:0048863 | stem cell differentiation | 4/22 | 0.0018 | 4 |
| BP | GO:0007492 | endoderm development | 3/22 | 0.001837 | 3 |
| BP | GO:0035050 | embryonic heart tube development | 3/22 | 0.002252 | 3 |
| BP | GO:0009913 | epidermal cell differentiation | 4/22 | 0.002377 | 4 |
| BP | GO:0048820 | hair follicle maturation | 2/22 | 0.002377 | 2 |
| BP | GO:0001656 | metanephros development | 3/22 | 0.002504 | 3 |
| BP | GO:0001942 | hair follicle development | 3/22 | 0.002504 | 3 |
| BP | GO:0042448 | progesterone metabolic process | 2/22 | 0.002587 | 2 |
| BP | GO:0009953 | dorsal/ventral pattern formation | 3/22 | 0.002598 | 3 |
| BP | GO:0022404 | molting cycle process | 3/22 | 0.002598 | 3 |
| BP | GO:0022405 | hair cycle process | 3/22 | 0.002598 | 3 |
| BP | GO:0048762 | mesenchymal cell differentiation | 4/22 | 0.002604 | 4 |
| BP | GO:0098773 | skin epidermis development | 3/22 | 0.002604 | 3 |
| BP | GO:0001657 | ureteric bud development | 3/22 | 0.002612 | 3 |
| BP | GO:0048704 | embryonic skeletal system morphogenesis | 3/22 | 0.002612 | 3 |
| BP | GO:0072163 | mesonephric epithelium development | 3/22 | 0.002622 | 3 |
| BP | GO:0072164 | mesonephric tubule development | 3/22 | 0.002622 | 3 |
| BP | GO:0003007 | heart morphogenesis | 4/22 | 0.002774 | 4 |
| BP | GO:0021549 | cerebellum development | 3/22 | 0.002774 | 3 |
| BP | GO:0002076 | osteoblast development | 2/22 | 0.002774 | 2 |
| BP | GO:0001823 | mesonephros development | 3/22 | 0.002808 | 3 |
| BP | GO:0061323 | cell proliferation involved in heart morphogenesis | 2/22 | 0.00298 | 2 |
| BP | GO:0072079 | nephron tubule formation | 2/22 | 0.00298 | 2 |
| BP | GO:0090596 | sensory organ morphogenesis | 4/22 | 0.003124 | 4 |
| BP | GO:0006700 | C21-steroid hormone biosynthetic process | 2/22 | 0.003227 | 2 |
| BP | GO:0022037 | metencephalon development | 3/22 | 0.003317 | 3 |
| BP | GO:0032526 | response to retinoic acid | 3/22 | 0.00346 | 3 |
| BP | GO:0002062 | chondrocyte differentiation | 3/22 | 0.003512 | 3 |
| BP | GO:0072009 | nephron epithelium development | 3/22 | 0.003565 | 3 |
| BP | GO:0023019 | signal transduction involved in regulation of gene expression | 2/22 | 0.00369 | 2 |
| BP | GO:0042303 | molting cycle | 3/22 | 0.003724 | 3 |
| BP | GO:0042633 | hair cycle | 3/22 | 0.003724 | 3 |
| BP | GO:0032331 | negative regulation of chondrocyte differentiation | 2/22 | 0.003859 | 2 |
| BP | GO:0043968 | histone H2A acetylation | 2/22 | 0.003859 | 2 |
| BP | GO:0048562 | embryonic organ morphogenesis | 4/22 | 0.00402 | 4 |
| BP | GO:0043588 | skin development | 4/22 | 0.004028 | 4 |
| BP | GO:0001759 | organ induction | 2/22 | 0.004028 | 2 |
| BP | GO:0120255 | olefinic compound biosynthetic process | 2/22 | 0.004028 | 2 |
| BP | GO:0048593 | camera-type eye morphogenesis | 3/22 | 0.004166 | 3 |
| BP | GO:0060485 | mesenchyme development | 4/22 | 0.004166 | 4 |
| BP | GO:0032355 | response to estradiol | 3/22 | 0.004197 | 3 |
| BP | GO:0033233 | regulation of protein sumoylation | 2/22 | 0.004197 | 2 |
| BP | GO:0001702 | gastrulation with mouth forming second | 2/22 | 0.004497 | 2 |
| BP | GO:0034505 | tooth mineralization | 2/22 | 0.004805 | 2 |
| BP | GO:0045667 | regulation of osteoblast differentiation | 3/22 | 0.005441 | 3 |
| BP | GO:0003156 | regulation of animal organ formation | 2/22 | 0.005441 | 2 |
| BP | GO:0061037 | negative regulation of cartilage development | 2/22 | 0.005751 | 2 |
| BP | GO:0072073 | kidney epithelium development | 3/22 | 0.005751 | 3 |
| BP | GO:0035115 | embryonic forelimb morphogenesis | 2/22 | 0.005989 | 2 |
| BP | GO:0061311 | cell surface receptor signaling pathway involved in heart development | 2/22 | 0.005989 | 2 |
| BP | GO:0010669 | epithelial structure maintenance | 2/22 | 0.006324 | 2 |
| BP | GO:0009954 | proximal/distal pattern formation | 2/22 | 0.006947 | 2 |
| BP | GO:0031128 | developmental induction | 2/22 | 0.006947 | 2 |
| BP | GO:0110110 | positive regulation of animal organ morphogenesis | 2/22 | 0.006947 | 2 |
| BP | GO:0048754 | branching morphogenesis of an epithelial tube | 3/22 | 0.006977 | 3 |
| BP | GO:0048592 | eye morphogenesis | 3/22 | 0.007047 | 3 |
| BP | GO:0007519 | skeletal muscle tissue development | 3/22 | 0.007117 | 3 |
| BP | GO:0051098 | regulation of binding | 4/22 | 0.007258 | 4 |
| BP | GO:0030856 | regulation of epithelial cell differentiation | 3/22 | 0.007258 | 3 |
| BP | GO:0008207 | C21-steroid hormone metabolic process | 2/22 | 0.007672 | 2 |
| BP | GO:0010092 | specification of animal organ identity | 2/22 | 0.007672 | 2 |
| BP | GO:1905168 | positive regulation of double-strand break repair via homologous recombination | 2/22 | 0.007672 | 2 |
| BP | GO:0001837 | epithelial to mesenchymal transition | 3/22 | 0.007682 | 3 |
| BP | GO:0035136 | forelimb morphogenesis | 2/22 | 0.007892 | 2 |
| BP | GO:0045616 | regulation of keratinocyte differentiation | 2/22 | 0.007892 | 2 |
| BP | GO:0016573 | histone acetylation | 3/22 | 0.008115 | 3 |
| BP | GO:0060538 | skeletal muscle organ development | 3/22 | 0.008115 | 3 |
| BP | GO:0071392 | cellular response to estradiol stimulus | 2/22 | 0.008464 | 2 |
| BP | GO:0072210 | metanephric nephron development | 2/22 | 0.008464 | 2 |
| BP | GO:0051099 | positive regulation of binding | 3/22 | 0.008483 | 3 |
| BP | GO:0018393 | internal peptidyl-lysine acetylation | 3/22 | 0.008616 | 3 |
| BP | GO:0034243 | regulation of transcription elongation from RNA polymerase II promoter | 2/22 | 0.008616 | 2 |
| BP | GO:0120178 | steroid hormone biosynthetic process | 2/22 | 0.008616 | 2 |
| BP | GO:0048771 | tissue remodeling | 3/22 | 0.008645 | 3 |
| BP | GO:0006475 | internal protein amino acid acetylation | 3/22 | 0.008722 | 3 |
| BP | GO:0001709 | cell fate determination | 2/22 | 0.00919 | 2 |
| BP | GO:0042181 | ketone biosynthetic process | 2/22 | 0.00919 | 2 |
| BP | GO:0018394 | peptidyl-lysine acetylation | 3/22 | 0.009852 | 3 |
| BP | GO:0061138 | morphogenesis of a branching epithelium | 3/22 | 0.009933 | 3 |
| BP | GO:0001503 | ossification | 4/22 | 0.010231 | 4 |
| BP | GO:0007369 | gastrulation | 3/22 | 0.010568 | 3 |
| BP | GO:0035315 | hair cell differentiation | 2/22 | 0.010573 | 2 |
| BP | GO:0030857 | negative regulation of epithelial cell differentiation | 2/22 | 0.01181 | 2 |
| BP | GO:0001763 | morphogenesis of a branching structure | 3/22 | 0.01181 | 3 |
| BP | GO:0032330 | regulation of chondrocyte differentiation | 2/22 | 0.012171 | 2 |
| BP | GO:0010718 | positive regulation of epithelial to mesenchymal transition | 2/22 | 0.012952 | 2 |
| BP | GO:0072132 | mesenchyme morphogenesis | 2/22 | 0.012952 | 2 |
| BP | GO:0045637 | regulation of myeloid cell differentiation | 3/22 | 0.01303 | 3 |
| BP | GO:0016925 | protein sumoylation | 2/22 | 0.013255 | 2 |
| BP | GO:0006473 | protein acetylation | 3/22 | 0.01426 | 3 |
| BP | GO:0001541 | ovarian follicle development | 2/22 | 0.01426 | 2 |
| BP | GO:0001658 | branching involved in ureteric bud morphogenesis | 2/22 | 0.01426 | 2 |
| BP | GO:0009948 | anterior/posterior axis specification | 2/22 | 0.01426 | 2 |
| BP | GO:0032784 | regulation of DNA-templated transcription, elongation | 2/22 | 0.01426 | 2 |
| BP | GO:0045604 | regulation of epidermal cell differentiation | 2/22 | 0.014661 | 2 |
| BP | GO:0060348 | bone development | 3/22 | 0.015443 | 3 |
| BP | GO:0016570 | histone modification | 4/22 | 0.016477 | 4 |
| BP | GO:0006368 | transcription elongation from RNA polymerase II promoter | 2/22 | 0.016708 | 2 |
| BP | GO:0045453 | bone resorption | 2/22 | 0.016708 | 2 |
| BP | GO:0060675 | ureteric bud morphogenesis | 2/22 | 0.016708 | 2 |
| BP | GO:0048738 | cardiac muscle tissue development | 3/22 | 0.016712 | 3 |
| BP | GO:0045670 | regulation of osteoclast differentiation | 2/22 | 0.016804 | 2 |
| BP | GO:0072171 | mesonephric tubule morphogenesis | 2/22 | 0.016804 | 2 |
| BP | GO:2000781 | positive regulation of double-strand break repair | 2/22 | 0.016804 | 2 |
| BP | GO:0030858 | positive regulation of epithelial cell differentiation | 2/22 | 0.016902 | 2 |
| BP | GO:0042446 | hormone biosynthetic process | 2/22 | 0.016902 | 2 |
| BP | GO:0045682 | regulation of epidermis development | 2/22 | 0.016902 | 2 |
| BP | GO:0048645 | animal organ formation | 2/22 | 0.016902 | 2 |
| BP | GO:0001649 | osteoblast differentiation | 3/22 | 0.017208 | 3 |
| BP | GO:0032835 | glomerulus development | 2/22 | 0.017208 | 2 |
| BP | GO:0014706 | striated muscle tissue development | 3/22 | 0.01852 | 3 |
| BP | GO:0010569 | regulation of double-strand break repair via homologous recombination | 2/22 | 0.018755 | 2 |
| BP | GO:0042698 | ovulation cycle | 2/22 | 0.018755 | 2 |
| BP | GO:0045911 | positive regulation of DNA recombination | 2/22 | 0.018755 | 2 |
| BP | GO:0061035 | regulation of cartilage development | 2/22 | 0.018755 | 2 |
| BP | GO:0043966 | histone H3 acetylation | 2/22 | 0.01948 | 2 |
| BP | GO:0050810 | regulation of steroid biosynthetic process | 2/22 | 0.01948 | 2 |
| BP | GO:0072078 | nephron tubule morphogenesis | 2/22 | 0.01948 | 2 |
| BP | GO:0043543 | protein acylation | 3/22 | 0.019758 | 3 |
| BP | GO:0043967 | histone H4 acetylation | 2/22 | 0.019787 | 2 |
| BP | GO:0045669 | positive regulation of osteoblast differentiation | 2/22 | 0.020094 | 2 |
| BP | GO:0072088 | nephron epithelium morphogenesis | 2/22 | 0.020094 | 2 |
| BP | GO:0051054 | positive regulation of DNA metabolic process | 3/22 | 0.020389 | 3 |
| BP | GO:0061333 | renal tubule morphogenesis | 2/22 | 0.020937 | 2 |
| BP | GO:0072028 | nephron morphogenesis | 2/22 | 0.021362 | 2 |
| BP | GO:0006354 | DNA-templated transcription, elongation | 2/22 | 0.023329 | 2 |
| BP | GO:0031016 | pancreas development | 2/22 | 0.023329 | 2 |
| BP | GO:0007548 | sex differentiation | 3/22 | 0.023883 | 3 |
| BP | GO:0014032 | neural crest cell development | 2/22 | 0.024204 | 2 |
| BP | GO:2000243 | positive regulation of reproductive process | 2/22 | 0.02522 | 2 |
| BP | GO:1901216 | positive regulation of neuron death | 2/22 | 0.026249 | 2 |
| BP | GO:0014031 | mesenchymal cell development | 2/22 | 0.026417 | 2 |
| BP | GO:0046849 | bone remodeling | 2/22 | 0.026417 | 2 |
| BP | GO:0048864 | stem cell development | 2/22 | 0.026417 | 2 |
| BP | GO:0045638 | negative regulation of myeloid cell differentiation | 2/22 | 0.027761 | 2 |
| BP | GO:0072080 | nephron tubule development | 2/22 | 0.027761 | 2 |
| BP | GO:0090398 | cellular senescence | 2/22 | 0.027761 | 2 |
| BP | GO:0060993 | kidney morphogenesis | 2/22 | 0.028812 | 2 |
| BP | GO:0045739 | positive regulation of DNA repair | 2/22 | 0.029117 | 2 |
| BP | GO:0061326 | renal tubule development | 2/22 | 0.029117 | 2 |
| BP | GO:0014033 | neural crest cell differentiation | 2/22 | 0.029571 | 2 |
| BP | GO:0030316 | osteoclast differentiation | 2/22 | 0.030027 | 2 |
| BP | GO:0000079 | regulation of cyclin-dependent protein serine/threonine kinase activity | 2/22 | 0.030941 | 2 |
| BP | GO:0060349 | bone morphogenesis | 2/22 | 0.030941 | 2 |
| BP | GO:1904029 | regulation of cyclin-dependent protein kinase activity | 2/22 | 0.032641 | 2 |
| BP | GO:0010717 | regulation of epithelial to mesenchymal transition | 2/22 | 0.033106 | 2 |
| BP | GO:0060562 | epithelial tube morphogenesis | 3/22 | 0.033277 | 3 |
| BP | GO:0035282 | segmentation | 2/22 | 0.033407 | 2 |
| BP | GO:0019218 | regulation of steroid metabolic process | 2/22 | 0.033872 | 2 |
| BP | GO:0008585 | female gonad development | 2/22 | 0.034338 | 2 |
| BP | GO:0021761 | limbic system development | 2/22 | 0.036086 | 2 |
| BP | GO:0046545 | development of primary female sexual characteristics | 2/22 | 0.036558 | 2 |
| BP | GO:2000779 | regulation of double-strand break repair | 2/22 | 0.03703 | 2 |
| BP | GO:0002761 | regulation of myeloid leukocyte differentiation | 2/22 | 0.041511 | 2 |
| BP | GO:0007178 | transmembrane receptor protein serine/threonine kinase signaling pathway | 3/22 | 0.043912 | 3 |
| BP | GO:1903706 | regulation of hemopoiesis | 3/22 | 0.044684 | 3 |
| BP | GO:0001704 | formation of primary germ layer | 2/22 | 0.044849 | 2 |
| BP | GO:0022612 | gland morphogenesis | 2/22 | 0.044849 | 2 |
| BP | GO:0001838 | embryonic epithelial tube formation | 2/22 | 0.04513 | 2 |
| BP | GO:0046660 | female sex differentiation | 2/22 | 0.04513 | 2 |
| BP | GO:0003206 | cardiac chamber morphogenesis | 2/22 | 0.046324 | 2 |
| BP | GO:2000027 | regulation of animal organ morphogenesis | 2/22 | 0.04802 | 2 |
| BP | GO:2001022 | positive regulation of response to DNA damage stimulus | 2/22 | 0.04802 | 2 |
| BP | GO:0001701 | in utero embryonic development | 3/22 | 0.048085 | 3 |
| BP | GO:0042692 | muscle cell differentiation | 3/22 | 0.048085 | 3 |
| BP | GO:0000018 | regulation of DNA recombination | 2/22 | 0.048788 | 2 |
| BP | GO:0030099 | myeloid cell differentiation | 3/22 | 0.049002 | 3 |
| BP | GO:0007498 | mesoderm development | 2/22 | 0.049062 | 2 |
| CC | GO:0005667 | transcription regulator complex | 9/22 | 1.18E-07 | 9 |
| CC | GO:0000812 | Swr1 complex | 2/22 | 0.002893 | 2 |
| CC | GO:1990907 | beta-catenin-TCF complex | 2/22 | 0.002893 | 2 |
| CC | GO:0090575 | RNA polymerase II transcription regulator complex | 4/22 | 0.002893 | 4 |
| CC | GO:0035267 | NuA4 histone acetyltransferase complex | 2/22 | 0.005552 | 2 |
| CC | GO:0043189 | H4/H2A histone acetyltransferase complex | 2/22 | 0.005552 | 2 |
| CC | GO:0097346 | INO80-type complex | 2/22 | 0.005552 | 2 |
| CC | GO:1902562 | H4 histone acetyltransferase complex | 2/22 | 0.011548 | 2 |
| CC | GO:0032993 | protein-DNA complex | 3/22 | 0.020612 | 3 |
| CC | GO:0000118 | histone deacetylase complex | 2/22 | 0.029624 | 2 |
| CC | GO:0070603 | SWI/SNF superfamily-type complex | 2/22 | 0.033759 | 2 |
| CC | GO:0000123 | histone acetyltransferase complex | 2/22 | 0.033759 | 2 |
| CC | GO:1904949 | ATPase complex | 2/22 | 0.033759 | 2 |
| CC | GO:0031248 | protein acetyltransferase complex | 2/22 | 0.03537 | 2 |
| CC | GO:1902493 | acetyltransferase complex | 2/22 | 0.03537 | 2 |
| MF | GO:0001228 | DNA-binding transcription activator activity, RNA polymerase II-specific | 9/22 | 2.95E-08 | 9 |
| MF | GO:0001216 | DNA-binding transcription activator activity | 9/22 | 2.95E-08 | 9 |
| MF | GO:0140297 | DNA-binding transcription factor binding | 9/22 | 2.95E-08 | 9 |
| MF | GO:0061629 | RNA polymerase II-specific DNA-binding transcription factor binding | 8/22 | 5.35E-08 | 8 |
| MF | GO:0035035 | histone acetyltransferase binding | 2/22 | 0.003804 | 2 |
| MF | GO:0005109 | frizzled binding | 2/22 | 0.008714 | 2 |
| MF | GO:1990841 | promoter-specific chromatin binding | 2/22 | 0.018255 | 2 |
| MF | GO:0003712 | transcription coregulator activity | 4/22 | 0.018255 | 4 |
| MF | GO:0046332 | SMAD binding | 2/22 | 0.02411 | 2 |
| MF | GO:0008013 | beta-catenin binding | 2/22 | 0.026258 | 2 |
| MF | GO:0001227 | DNA-binding transcription repressor activity, RNA polymerase II-specific | 3/22 | 0.030545 | 3 |
| MF | GO:0001217 | DNA-binding transcription repressor activity | 3/22 | 0.030545 | 3 |

**Supplementary Table 1.** The functions of PITX1, PITX2, and coexpression genes were predicted by the GO analysis.

| ID | Description | GeneRatio | p.adjust | Count |
| --- | --- | --- | --- | --- |
| hsa05166 | Human T-cell leukemia virus 1 infection | 5/15 | 0.001987 | 5 |
| hsa04390 | Hippo signaling pathway | 4/15 | 0.003009 | 4 |
| hsa05217 | Basal cell carcinoma | 3/15 | 0.003009 | 3 |
| hsa04310 | Wnt signaling pathway | 4/15 | 0.003009 | 4 |
| hsa04916 | Melanogenesis | 3/15 | 0.008722 | 3 |
| hsa04550 | Signaling pathways regulating pluripotency of stem cells | 3/15 | 0.015005 | 3 |
| hsa05224 | Breast cancer | 3/15 | 0.015005 | 3 |
| hsa05226 | Gastric cancer | 3/15 | 0.015005 | 3 |
| hsa05165 | Human papillomavirus infection | 4/15 | 0.015005 | 4 |
| hsa04934 | Cushing syndrome | 3/15 | 0.015005 | 3 |
| hsa05225 | Hepatocellular carcinoma | 3/15 | 0.017146 | 3 |
| hsa05205 | Proteoglycans in cancer | 3/15 | 0.027499 | 3 |
| hsa04912 | GnRH signaling pathway | 2/15 | 0.054676 | 2 |
| hsa04928 | Parathyroid hormone synthesis, secretion and action | 2/15 | 0.065141 | 2 |
| hsa04371 | Apelin signaling pathway | 2/15 | 0.094866 | 2 |
| hsa05418 | Fluid shear stress and atherosclerosis | 2/15 | 0.094866 | 2 |
| hsa05010 | Alzheimer disease | 3/15 | 0.104406 | 3 |
| hsa04150 | mTOR signaling pathway | 2/15 | 0.104406 | 2 |
| hsa04950 | Maturity onset diabetes of the young | 1/15 | 0.138492 | 1 |
| hsa05202 | Transcriptional misregulation in cancer | 2/15 | 0.138492 | 2 |
| hsa04510 | Focal adhesion | 2/15 | 0.140135 | 2 |
| hsa05022 | Pathways of neurodegeneration - multiple diseases | 3/15 | 0.140135 | 3 |
| hsa05216 | Thyroid cancer | 1/15 | 0.166335 | 1 |
| hsa04913 | Ovarian steroidogenesis | 1/15 | 0.217118 | 1 |
| hsa04340 | Hedgehog signaling pathway | 1/15 | 0.226574 | 1 |
| hsa05213 | Endometrial cancer | 1/15 | 0.226574 | 1 |
| hsa04917 | Prolactin signaling pathway | 1/15 | 0.24656 | 1 |
| hsa04520 | Adherens junction | 1/15 | 0.24656 | 1 |
| hsa04115 | p53 signaling pathway | 1/15 | 0.24656 | 1 |
| hsa05100 | Bacterial invasion of epithelial cells | 1/15 | 0.24656 | 1 |
| hsa05412 | Arrhythmogenic right ventricular cardiomyopathy | 1/15 | 0.24656 | 1 |
| hsa04151 | PI3K-Akt signaling pathway | 2/15 | 0.24656 | 2 |
| hsa05210 | Colorectal cancer | 1/15 | 0.258496 | 1 |
| hsa04350 | TGF-beta signaling pathway | 1/15 | 0.2723 | 1 |
| hsa05215 | Prostate cancer | 1/15 | 0.2723 | 1 |
| hsa04933 | AGE-RAGE signaling pathway in diabetic complications | 1/15 | 0.2723 | 1 |

**Supplementary Table 2.** The functions of PITX1, PITX2, and coexpression genes were predicted by the KEGG analysis.
